# Effects of Caffeinated Chewing Gum on Exercise Performance and Physiological Responses: A Systematic Review

**DOI:** 10.3390/nu16213611

**Published:** 2024-10-24

**Authors:** Chia-Cheng Yang, Meng-Hung Hsieh, Chien-Chang Ho, Ya-Hui Chang, Yi-Jie Shiu

**Affiliations:** 1 Department of Physical Education, National Taichung University of Education, Taichung 403, Taiwan; muchyang@mail.ntcu.edu.tw; 2 Department of Physical Education, Tunghai University, Taichung 407, Taiwan; meng.hung791118@gmail.com; 3 Department of Physical Education, Fu Jen Catholic University, New Taipei City 242, Taiwan; 093703@mail.fju.edu.tw; 4 Graduate Program in Department of Exercise Health Science, National Taiwan University of Sport, Taichung 404, Taiwan; yahuichang36@gmail.com; 5 Department of Physical Education and Sport Sciences, National Taiwan Normal University, Taipei 106, Taiwan

**Keywords:** sympathetic activation, ergogenic aids, nutrition

## Abstract

Background: Caffeine intake in the form of chewing gum is characterized by rapid absorption and utilization. Objectives: The purpose of this study was to investigate the effects of caffeinated chewing gum on exercise performance and physiological responses in a systematic review. Methods: All articles were searched using the PubMed and Scopus databases to include articles published up to June 2024, following the Preferred Reporting Items for Systematic Evaluation and Meta-Analysis (PRISMA) protocol. Results: Thirty-two studies were finally included. Most studies have found that pre-exercise caffeinated chewing gum supplementation is effective in improving endurance, repetitive sprinting, lower limb strength, and sport-specific performance, as well as lowering rating of perceived exertion (RPE) or fatigue index even with lower dosages of caffeine. Sympathetic activation may be one of the mechanisms by which caffeinated chewing gum affects athletic performance. No significant effect on energy metabolism indicators (blood glucose, blood lactate, free fatty acids) was found. In addition, two studies found that caffeinated chewing gum reduced or maintained cortisol levels and increased testosterone levels. However, caffeinated chewing gum intake does not have an impact on catecholamines and β-endorphins. There have been inconsistent results for explosive performance, agility performance, and pain perception. Only a few studies have examined balance performance. In conclusion, a low dose of caffeine (100–300 mg or 2–4 mg/kg) in the form of chewing gum is rapidly absorbed and utilized, positively impacting most exercise and physiological performance. Conclusions: Future studies should also consider the performance variables of agility, pain perception, and explosive performance to gain a more comprehensive understanding of the effects of caffeinated chewing gum on sympathetic activation and exercise performance.

## 1. Introduction

**Figure 1:**
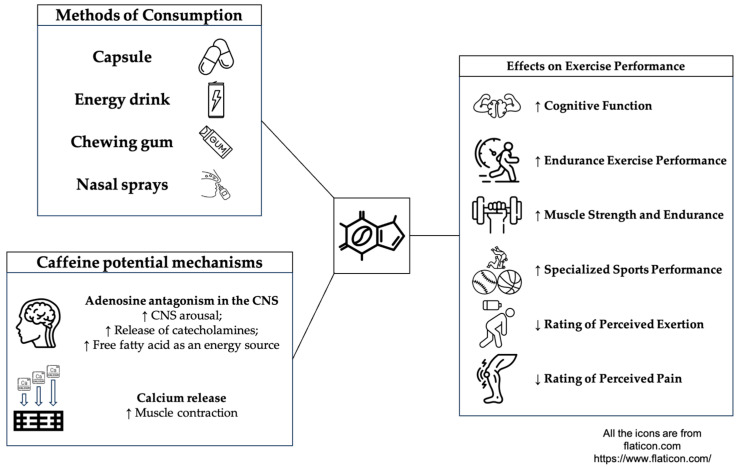
Mechanism of caffeine action. ↑ increased, ↓ decreased.

Caffeine is a xanthine alkaloid compound found in about 60 plant species and many foods. Because of its easy availability and stimulant properties, caffeine is considered the most widely used natural central nervous system (CNS) stimulant in the world. Since the World Anti-Doping Agency (WADA) removed caffeine from the doping list of banned sports drugs in 2004, most athletes consume caffeine during training or competition to enhance performance [1,2]. Caffeine has three main mechanisms of action: (i) antagonizing adenosine, (ii) stimulating catecholamine release, and (iii) enhancing the ability of the sarcoplasmic reticulum to release calcium [3] (Figure 1). Caffeine’s primary mechanism of action is adenosine antagonism in the CNS, resulting in an increase in CNS arousal. Caffeine has a similar structure to adenosine and antagonizes adenosine. Adenosine is found in the central nervous system and throughout the body, and when adenosine binds to the receptor, it produces a perception of fatigue. Caffeine crosses the blood–brain barrier (BBB) and binds to adenosine receptors, reducing fatigue perception [4]. In addition, when caffeine binds to adenosine receptors, it stimulates the release of catecholamines, including epinephrine (EPI) and norepinephrine (NE), resulting in a Fight or Flight response, increased alertness, and activation of the autonomic nervous system (ANS) [5]. Increased epinephrine levels stimulate the release of free fatty acid (FFA) as an energy source during exercise and enhance endurance performance [6,7]. The other caffeine potential mechanisms are that caffeine alters the release or uptake of calcium in the sarcoplasmic reticulum to enhance muscle performance [8,9]. It also enhances excitation-contraction coupling by increasing the activity of the sodium-potassium pump, which promotes muscle performance and reduces performance decline during endurance exercise [5,8].

Caffeine can be consumed in various ways, commonly in the form of powders, capsules, and energy drinks [10,11]. In recent years, there have been many additional methods of intake, such as caffeinated chewing gum [12,13], gel [14], nasal spray [15], and mouth rinsing [16]. Among them, ingestion through chewing gum can effectively release caffeine (80%) within a short period (5–10 min) and allow it to enter the systemic circulation through oral mucosa absorption, while part of the caffeine enters the gastrointestinal tract with saliva to form secondary absorption [17,18]. Caffeine intake through chewing gum can be beneficial to those who need to absorb caffeine quickly, as it reduces water intake and gastrointestinal discomfort compared with the traditional method [19].

Caffeine intake through chewing gum has been studied since 2002 [17]. Over the past decade, caffeinated gum consumption has been effective in enhancing aerobic exercise [20,21], anaerobic exercise [22], resistance training [13], and specialized performance [12].

Given the conflicting findings in studies on caffeinated gum and exercise performance, this systematic review aims to evaluate past research to clarify the effects of caffeinated chewing gum on exercise performance, with the hypothesis that it may have the advantage of rapid absorption and can effectively enhance exercise performance. Additionally, the relationship between caffeinated gum supplementation, the autonomic nervous system, and exercise outcomes will be explored. Insights into the effectiveness of caffeinated chewing gum for healthy individuals will be provided, along with a highlight of areas for future research.

## 2. Materials and Methods

### 2.1. Design

This systematic review was conducted following the guidelines and recommendations outlined in the Preferred Reporting Items for Systematic Evaluation and Meta-Analysis (PRISMA) 2020 statement [23].

### 2.2. Search Strategy

The study was conducted on 30 June 2024, with a literature search performed for studies published up to 30 June 2024. Two researchers (Shiu and Yang) worked independently, coordinating a step-by-step data search in the electronic databases PubMed and Scopus using the following keywords: “caffeine or caffeinated” and “gum or chewing or chewing gum”. The article type was initially filtered using “Randomized Controlled Trials”. Unpublished documents, review studies, conference abstracts, theses, and book chapters were excluded. To minimize publication bias, searches were not restricted by language or publication date, and automatic page translation to English was used. Additionally, manual cross-referencing was conducted on the reference lists of relevant articles to identify other pertinent studies.

### 2.3. Eligibility Criteria

In the present study, the inclusion criteria of articles were defined according to the Population Intervention Comparison Outcome Study Design (PICOS) principle [23]. Eligible articles included: P-Healthy participants, I-caffeine chewing gum, C-Placebo, O-at least one assessment of exercise performance or exercise physiological response, S-single or double-blind study design. This study excluded: non-healthy adults or participants with medical conditions, studies with sleep deprivation or sleep restriction interventions, studies assessing only cognitive function (including memory, work capacity, and level of arousal), lack of placebo trials, studies without a single caffeine chewing gum intervention, and studies lacking an exercise intervention.

### 2.4. Data Extraction

All search results were imported into the bibliographic management software EndNote (EndNote 20, Thomson Reuters, NY, USA), where duplicate articles were removed. Authors Shiu and Yang then manually screened the relevance of the remaining articles based on predefined inclusion criteria, evaluating the article titles and abstracts. Any differences in article selection were resolved through discussion with a third researcher (Ho). The results will be reported according to the PRISMA guidelines.

The data extraction process was carried out independently by Shiu and Yang, and data were collected from the included articles using an Excel sheet, which contained the following information: (i) first author and year of publication; (ii) study design; (iii) participant characteristics (including sample size, gender, age, and level of physical activity); (iv) caffeinated chewing gum intake dose; (v) chewing duration; (vi) intake timing relative to the onset of exercise; and (vii) key findings in the caffeinated chewing gum group versus the placebo group. If there was disagreement between the two researchers regarding data extraction, this was resolved through discussion with a third author.

### 2.5. Methodological Quality Assessment

The methodological quality of the included studies was evaluated using the Cochrane risk of bias tool [24]. Additionally, the Quality Assessment of Controlled Intervention Studies (QACIS) was employed to further assess the studies’ methodological quality. Each assessment item was rated as 1 (meets the criteria), 0 (does not meet the criteria), or N/A (not applicable). The final score was calculated by summing the scores of the evaluated items and dividing by the total number of items, resulting in a percentage. Two authors (Shiu and Yang) independently reviewed the studies’ quality, resolving any disagreements through discussion until a consensus was reached (Tables S1 and S2). Prior to achieving consensus, the Cohen’s kappa score was determined to be 0.71, indicating a substantial level of interrater reliability. Although the NHLBI tool does not provide a specific threshold for quality scores, the studies were classified according to general assessment guidelines as having low (≤50%), good (51–75%), or excellent (>75%) methodological quality [25,26].

## 3. Results

### 3.1. Search Results

**Figure 2:**
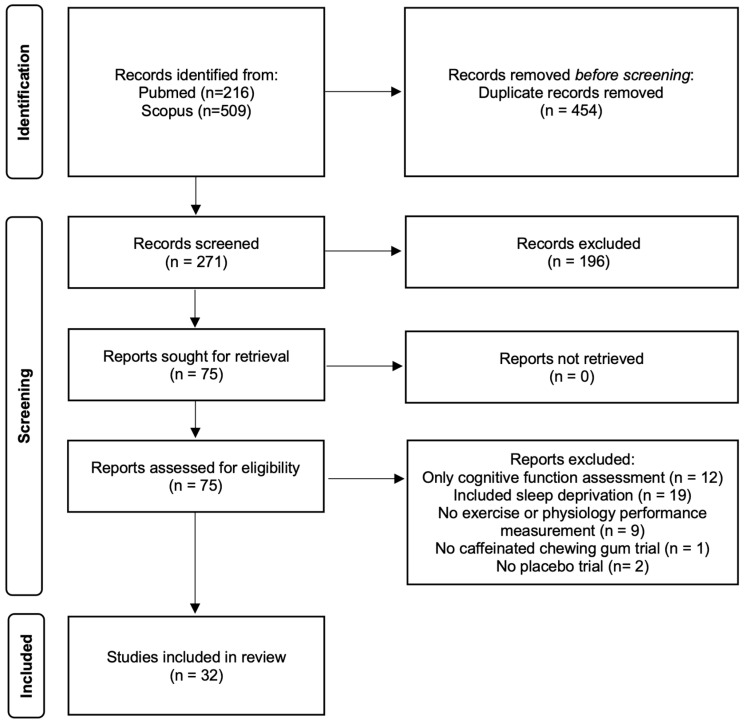
Search strategy and study selection process using PRISMA guidelines [23].

A total of 725 published articles were identified. Of these, 650 were excluded, leaving 75 articles for possible inclusion. 43 studies were excluded, including those that only assessed cognitive function (n = 12), included sleep deprivation in the experimental protocol (n = 19), did not include exercise performance or physiological responses before and after exercise (n = 9), lacked caffeine alone (n = 1), and lacked placebo trial (n = 2). 32 studies were included (Figure 2). Studies eligible for inclusion were published between 2010 and 2024.

### 3.2. Characteristics of the Included Studies

Table S3 presents the characteristics of the study population and caffeinated chewing gum ingestion protocol. A total of 32 studies were included in this systematic review, with 494 subjects, of which 370 were male and 124 were female. 19 studies included only male subjects, 3 included only female subjects, and 10 included both male and female subjects. The physical activity levels of the subjects included untrained subjects, healthy adults, trained subjects, collegiate athletes, and professional athletes. 12 studies included subjects with low-to-moderate habitual caffeine intake (<300 mg/day) only, 10 studies included subjects with mild-to-high habitual caffeine intake (>300 mg/day), and 13 studies did not report on the screening of caffeine habits or whether the subjects had a caffeine intake habit.

### 3.3. Endurance Exercise Performance

Caffeine has a beneficial effect on endurance exercise performance. Table 1 presents the information and outcomes related to this performance. The effects of caffeinated chewing gum on endurance were examined in 15 studies; among them, 10 indicated that consuming caffeinated gum before and during exercise had positive effects, such as reduced completion time, increased power output, and improved time to exhaustion (TTE) or distance. In contrast, the remaining 5 studies found no significant impact of either single or repeated chewing of caffeinated gum on endurance performance, including 2 studies specifically focused on middle- and long-distance events [27,28,29,30,31,32]. Ryan et al. (2013) investigated the effects of chewing gum containing 300 mg of caffeine for 5 min at different time points on cycling time trial performance. The results showed that caffeinated chewing gum ingestion 5 min before exercise significantly reduced the finish time (CAF: 38.7 ± 1.2, PLA: 40.7 ± 1.2 min, *p* = 0.023) [27]. Lane et al. (2014) found that chewing gum containing 3 mg/kg of caffeine for 10 min prior to exercise significantly reduced the completion time of cyclists or triathletes in a simulated cycling time trial (males: 1.3%, *p* < 0.001, females: 1.6%, *p* < 0.001) as well as enhancing significant mean output power (CAF. 260 ± 58 W, PLA: 250 ± 57 W, *p* < 0.001) (improvement 4.0 ± 1.7%MAP, *p* < 0.001) [32]. Oberlin-Brown et al. (2016) found that chewing gum containing 50 mg of caffeine for 5 min every 25% of distance during time trial exercise did not have significant effects on both time trial performance and overall mean power output, but did improve later stage cycling mean power output (for 10–15 km: CAF: 263 ± 39, PLA: 259 ± 35 W, ES: 0.09; for 15–20 km: CAF: 284 ± 42, PLA: 273 ± 41 W, ES: 0.24) [33]. Ranchordas et al. (2018) found that chewing gum containing 200 mg of caffeine intake for 5 min before exercise significantly enhanced soccer players’ YO-YO IR1 test covered distance (CAF: 1754 ± 156, PLA: 1719 ± 139 m, *p* = 0.016, ES: 0.24; enhanced by 2%) [34]. Ranchordas et al. (2019) found that consuming chewing gum containing 200 mg of caffeine for 5 min before exercise significantly enhanced rugby players YO-YO IR2 test covered distance (CAF: 426 ± 105; PLA: 372 ± 91 m, *p* = 0.010, ES. 0.55; enhanced by 14.5%) [35]. Daneshfar et al. (2020) found that pre-exercise chewing of gum containing 300 mg of caffeine significantly improved time trial performance (*p* = 0.001, ES: 0.71), peak power to weight ratio (*p* = 0.001, ES: 0.79), and maximum power to weight ratio (*p* = 0.001, ES: 0.80) in motocross cyclists [36]. Whalley et al. (2020) found that intake of chewing gum containing 3 or 4.5 mg/kg caffeine for 15 min before exercise significantly improved the performance of amateur runners in a 5 km running time trial (Gum: 0.9% ± 1.4%, *p* > 0.005) [37]. Dittrich et al. (2021) found that intake of chewing gum containing 300 mg of caffeine for 5 min before exercise significantly increased trained endurance runners’ TTE at 50% maximal aerobic speed (CAF: 40.60 ± 8.53; PLA. 33.23 ± 7.41 min, *p* < 0.001), and total distance covered (CAF: 10.36 ± 2.19; PLA: 8.45 ± 1.73 km, *p* < 0.001) [21]. Farmani et al. (2024) found that chewing gum containing 200 mg (for 65 kg↓) or 300 mg (for 65 kg↑) of caffeine consumption for 10 min before exercise significantly increased table tennis players’ TTE performance (CAF: 12.26 ± 1.30, PLA: 11.58 ± 1.22 min, *p* < 0.001) and increased VO2 at VT1 (CAF: 25.89 ± 2.27, PLA: 24.26 ± 1.57 L/min, *p* = 0.004) and VO2 at RCP (CAF: 31.35 ± 2.92, PLA: 27.93 ± 2.64 L/min, *p* < 0.001); yet, there was no significant effect on VO2max (CAF: 50.11 ± 5.14, PLA: 50.50 ± 5.98 mL/min/kg, *p* = 0.877) [20]. Lynn et al. (2024) found that the intake of chewing gum containing 300 mg of caffeine for 5 min and for 30 min before exercise reduced habitual caffeine consumption of recreational runners in a 5 km park run time trial finish time (17.28 s, *p* = 0.01). There were no significant differences in split time (*p* = 0.06) and running pacing (*p* = 0.21) [38].

### 3.4. Sprint Performance

Caffeine supplementation has been found to enhance sprinting performance and attenuate performance decline following repeated sprints. Table 2 provides information and outcomes on sprint performance. A total of 4 studies investigated the effects of caffeinated gum on single sprint performance. Only one study showed a positive benefit of chewing caffeinated gum. Liu et al. (2024) found that consuming chewing gum containing 3 mg/kg caffeine for 10 min before exercise significantly improved 20 m sprint performance in basketball athletes compared to the placebo, both in split time (0–10 m, *p* = 0.045, ES: 0.94; 10–20 m, *p* = 0.019, ES: 0.70) and total time (CAF: 2.94 ± 1.12 s, PLA: 3.13 ± 0.10 s, *p* < 0.001, ES: 1.8) [12]. Three other studies found no significant effects of caffeinated gum chewing on short-distance sprint performance (5–30 m), including total sprint time and acceleration performance [34,41,42].

Six studies have examined the effects of caffeinated gum on repetitive sprinting performance. Four of these studies found that chewing caffeinated chewing gum may enhance repetitive sprinting performance. Paton et al. (2010) found that chewing gum containing 240 mg of caffeine for 5 min during the repetitive sprint recovery period significantly reduced mean power output decline (5.4%, Cohen’s d: 0.25) [43]. Another study by Paton et al. (2015) found that chewing gum containing 300 mg (for males) or 200 mg (for females) of caffeine for 5 min during exercise significantly increased follow-up repetitive mean and peak sprint power output [40]. Evans et al. (2018) found that chewing gum containing 200 mg of caffeine consumption for 10 min before exercise significantly reduced low habitual caffeine intake for team sport athletes in the 40 m maximal shuttle run test decrement (CAF: 5.33 ± 3.12%, PLA: 6.53 ± 2.91%, *p* = 0.049, ES: 0.33). Nevertheless, there were no significant effects on participants with moderate-to-high habitual intake. Moreover, caffeinated chewing gum did not significantly improve the total time, fastest sprint time, or slowest sprint time performance in the repetitive sprints [44]. Liu et al. (2024) found that chewing gum containing 3 mg/kg for 10 min before exercise significantly increased basketball players’ mini power (CAF: 1234.44 ± 75.7, PLA: 1553.90 ± 35.9, *p* = 0.008, ES: 1.35) and mini power per weight (CAF: 16.30 ± 2.1, PLA: 1553.90 ± 35.9, *p* = 0.008, ES: 1.35) during running-base anaerobic sprint test performance, but had no significant effects on peak power or peak power per weight [12]. Two other studies found that chewing caffeinated gum before or during exercise had no significant effects on repetitive sprint completion time [35,45]; in spite of that, one of them discovered it was able to reduce fatigue index during repetitive sprint performance [35].

### 3.5. Strength Performance

Caffeine is widely recognized for its potential to enhance physical performance, including strength training. The information and results regarding strength performance are provided in Table 3. Four studies have examined the effects of caffeinated chewing gum on upper limb muscle strength, with one of these studies demonstrating a positive benefit. Additionally, five studies investigated the impact of caffeinated gum on lower limb muscle performance, and most of them indicated a positive effect on lower extremity muscle function. Venier et al. (2019) found that chewing gum containing 300 mg of caffeine for 10 min before exercise significantly increased healthy adult bench press velocity at 50%, 75%, and 90% 1 RM compared with the placebo [22]. No significant effects were found on upper extremity muscle strength performance and handgrip strength improvement while chewing caffeinated chewing gum in two studies [46,47]. Similarly, one study showed no significant difference between caffeine chewing gum and a placebo on grip to exhaustion performance [48].

Venier et al. (2019) found that chewing gum containing 300 mg of caffeine for 10 min prior to exercise significantly increased healthy adult knee flexion and extensor isokinetic strength and power in different angular velocity, and peak power output on the rowing ergometer test (CAF: 667.5 ± 78.5; PLA: 635.9 ± 68.7 W, *p* = 0.006, ES: 0.41) [22]. Dittrich et al. (2021) found that chewing gum containing 300 mg of caffeine for 5 min before exercise significantly reduced the decline in MVC performance after a TTE trial compared to the placebo, but there were no significant differences in muscle activity or neuromuscular function tests during MVC [21]. Chen et al. (2023) found that chewing gum containing 200 mg of caffeine for 5 min before exercise significantly improved healthy adult Romanian deadlift performance on a flywheel inertial device compared to the placebo, including peak concentric power (*p* = 0.016, ES: 0.44), peak eccentric power (*p* = 0.005, ES: 0.55), average power (*p* = 0.013, ES: 0.43), and total work (*p* = 0.026, ES: 0.28), but there was no significant difference in average force (*p* = 0.063, ES: 0.50) [13]. Yildirim et al. (2023) found that chewing gum containing 200 mg of caffeine for 5 min before exercise significantly increased quadriceps strength (CAF200: 53.77 ± 5.77, PLA: 49.20 ± 7.20 kg, *p* = 0.032, ES: 0.70). Compared with the placebo, no significant effect was found on hamstring strength (CAF200: 26.81 ± 5.83, CAF100: 28.11 ± 6.12, PLA: 25.66 ± 3.49 kg, *p* = 0.251) [46]. Liu et al. (2024) found that chewing gum containing 3 mg/kg caffeine for 10 min before exercise significantly improved squat performance on a flywheel device in basketball players, as well as average power (*p* = 0.012, ES: 0.41), peak concentric power (*p* = 0.013, ES: 0.48), and peak eccentric power (*p* = 0.028, ES: 0.45) in comparison with the placebo [12].

### 3.6. Explosive Power Performance

Caffeine’s role in enhancing explosive power performance is of significant interest in sports science, particularly in activities requiring rapid force production movements. The information and outcomes related to explosive power performance are presented in Table 4. Eight studies examined the effects of caffeinated gum on lower limb explosive performance, with four of these studies indicating that caffeinated gum positively influenced lower limb explosive strength. Ranchordas et al. (2018) found that chewing gum containing 200 mg of caffeine for 5 min before exercise significantly enhanced soccer players’ CMJ performance (CAF: 47.1 ± 3.4, PLA: 46.1 ± 3.2 cm, *p* = 0.008, ES: 0.30; enhanced by 2.2%) [34]. Another study by Ranchordas et al. (2019) found that chewing gum containing 200 mg of caffeine for 5 min prior to exercise significantly enhanced rugby players’ CMJ performance (CAF: 43.7 ± 7.6; PLA: 42.2 ± 6.2 cm, *p* = 0.044, ES: 0.22; enhanced by 3.6%) [35]. Venier et al. (2019) found that chewing gum containing 300 mg of caffeine for 10 min prior to exercise was effective in enhancing the performance of healthy adult CMJ (CAF: 36.4 ± 6.2; PLA: 34.8 ± 5.8 cm, *p* < 0.001, ES: 0.27) and SJ (CAF: 31.9 ± 6.0; PLA: 30.8 ± 5.3 cm, *p* = 0.023, ES: 0.21) performance [22]. In a study by Pirmohammadi et al. (2023), chewing gum containing 200 mg (for 65 kg↓) or 300 mg (for 65 kg↑) of caffeine for 10 min before exercise was found to improve table tennis player Sargent’s jump test performance (CAF: 1865.11 ± 452.00, PLA: 1689.55 ± 49.68 Watt, *p* = 0.001) [47]. However, the results of four studies showed that chewing caffeinated gum before exercise did not have a significant difference in single or consecutive vertical jump performance (including CMJ or SJ) [12,20,42,46].

### 3.7. Agility Performance

Agility is a crucial component of athletic performance, as it involves the ability to quickly change direction and respond to dynamic situations. The information and results on agility performance were collected and disclosed in Table 5. Four studies included agility performance measures; furthermore, one of the studies showed that chewing caffeinated gum was effective in improving agility performance. A study by Pirmohammadi et al. (2023) found that chewing gum containing 200 mg (for 65 kg↓) or 300 mg (for 65 kg↑) of caffeine for 10 min prior to exercise improved table tennis player performance on Edgren’s agility test (CAF: 24.38 ± 2.19, PLA: 23.22 ± 2.41 score, *p* = 0.002) compared to the placebo group [47]. Three other studies showed that caffeinated chewing gum did not improve agility performance, including for volleyball players or basketball players in the agility T-test [12,42] and for rugby players in the Illinois agility test [35].

### 3.8. Balance Performance

Balance is a vital aspect of overall athletic performance, influencing coordination and stability during physical activities. Among the studies, only one examined the effects of caffeinated gum on balance assessment. Cagin et al. (2024) found that the intake of chewing gum containing 5 mg/kg of caffeine 15 min prior to exercise did not have a significant difference on the Flamingo balance test compared to the placebo [41].

### 3.9. Cognitive Function and Reaction Performance

Cognitive functioning and reaction time play essential roles in athletic performance, as they influence decision-making and quick responses during competition. Three studies have examined the effects of caffeinated chewing gum on cognitive functioning and reaction time, two of which have shown positive benefits of caffeinated chewing gum. Bellar et al. (2012) found that chewing gum containing 100 mg of caffeine for 5 min prior to exercise significantly reduced the college shot putter’s pre-exercise and post-exercise simple reaction time task performance (pre-CAF: 0.306 ± 0.05 s, PLA: 0.317 ± 0.06 s, post-CAF: 0.316 ± 0.06 s, and post-CAF: 0.316 ± 0.06 s, *p* < 0.05, ES > 0.73) [49]. Cagin et al. (2024) found that chewing gum containing 5 mg/kg caffeine 15 min before exercise significantly reduced a sprinter’s 30 m sprint test reaction time performance compared to the placebo [41]. Another study indicated no significant effects of chewing caffeinated gum on cognitive performance reaction time, including the simple reaction time task and Stroop test, compared with the placebo during a simulated competition [45].

### 3.10. Specialized Sports Performance

More effects of caffeinated chewing gum on specialized sports performance were collected and organized (Table 6). Of seven eligible studies investigated in this field, in one study by Bellar et al. (2012), nine college shot putters chewed gum containing 100 mg of caffeine for 5 min before exercise and then performed six attempts. The results of the study showed that caffeinated chewing gum significantly improved first throw test performance (CAF: 9.62 ± 1.71, PLA: 9.05 ± 1.69 m, *p* = 0.050, effect size 0.996) and performance in the first group of three throws (*p* = 0.067, effect size 0.359) [49]. A study conducted by Filip-Stachnik et al. (2021) of nine experienced judoists who chewed gum containing either 200 mg or 400 mg of caffeine for 5 min before exercise followed by the Special Judo Fitness Test (SJFT) showed that the caffeinated chewing gum group did not significantly increase the total number of SJFT throws (CAF200: 62.22 ± 4.32; CAF400: 60.22 ± 4.08, PLA: 59.66 ± 4.15, *p* = 0.063) or affect the SJFT Index (trial, *p* = 0.099) compared to the placebo [50]. Another study by Filip-Stachnik et al. (2022) examined the effects of chewing gum containing 400 mg of caffeine for 5 min 15 min prior to exercise in 12 mild caffeine intake female volleyball players on attack jump, block jump, and game assessment performance. The results of the study showed that caffeinated chewing gum significantly increased their pre- and post-attack jump height (*p* = 0.024, pre-game CAF: 47.2 ± 7.3, PLA: 46.0 ± 7.9 cm, *p* = 0.032, post-game: CAF: 47.5 ± 7.5, PLA: 46.3 ± 8.3 cm, *p* = 0.022, ES: 0.15). Though caffeinated chewing gum significantly increased pre- and post-attack jump height, block jump height (pre-game CAF: 33.0 ± 4.5, PLA: 32.6 ± 5.7 cm, post-game: CAF: 34.7 ± 6.2, PLA: 34.8 ± 6.4 cm, *p* = 0.724), number of jumps during the game (CAF:52 ± 13; PLA: 47 ± 15 jumps, *p* = 0.273), along with volleyball-specific skills during the game (total points, total errors, service points, service errors, reception errors, negative reception errors, positive reception, perfect reception, and blocking points, *p* > 0.05) shown above indicated no significant differences [51]. A study by Kaszuba et al. (2022) of 12 volleyball players who chewed gum containing 200 mg (for female) or 300 mg (for male) of caffeine for 5 min and 15 min before exercise, followed by a series of volleyball skill tests, showed that caffeinated chewing gum significantly improved attack accuracy performance (CAF: 18 ± 3, PLA: 15 ± 4 points, *p* = 0.023, ES: 0.85), but had no significant effects on attack jump (CAF: 61.4 ± 14.9, PLA: 62.4 ± 13.9 cm, *p* = 0.342), block jump (CAF: 48.4 ± 10.6, PLA: 48.4 ± 11.6 cm, *p* = 0.995), standing attack speed (CAF: 82 ± 11, PLA: 79 ± 12 km/h, *p* = 0.274), attack speed (CAF: 85 ± 14, PLA: 81 ± 13 km/h, *p* = 0.119), service speed (CAF: 88 ± 14, PLA: 86 ± 13 km/h, *p* = 0.254), and serving accuracy (CAF: 12 ± 4, PLA: 10 ± 3 points, *p* = 0.140) [42]. Pirmohammadi et al. (2023) examined the effects of chewing gum containing 200 mg (for 65 kg↓) or 300 mg (for 65 kg↑) of caffeine on table tennis-related performance in 18 female table tennis players before exercise. Additionally, the results showed that the caffeinated chewing gum group significantly increased female table tennis players’ hand movement speed (CAF: 11.08 ± 1.27, PLA: 12.19 ± 1.43 n, *p* < 0.001), movement speed (CAF: 3.74 ± 0.22, PLA: 4.16 ± 4.0 s, *p* = 0.001), and table tennis related cognitive tests (CAF: 22.44 ± 1.5, PLA: 2.11 ± 1.84 n, *p* < 0.001). However, other table tennis-specific skills, including accuracy in eye-hand coordination test (CAF: 28 ± 1.45, PLA: 26.88 ± 2.21 n, *p* = 0.091), accuracy of service (CAF: 38.11 ± 4.82, PLA: 36.33 ± 4.02 score, *p* = 0.211), accuracy of forehand drive (CAF: 16.11 ± 2.58, PLA: 15.72 ± 1.99 score, *p* = 1.000), backhand- push performance (CAF: 28.05 ± 1.25, PLA: 28.30 ± 0.94 score, *p* = 0.318), and counter performance (CAF: 26.66 ± 1.87, PLA: 26.83 ± 1.33 n, *p* = 0.710) [47] were not significantly improved in the caffeine-gum group. Yildirim et al. (2023) investigated the effect of chewing gum containing 100 mg or 200 mg of caffeine on ball-kicking speed in 14 habitual caffeine intake soccer players 15 min before exercise. The results of the study showed that caffeinated chewing gum had no significant effect on ball-kicking speed (CAF200: 106.36 ± 5.79, CAF100: 107.80 ± 4.34, PLA: 106.74 ± 7.69 km/h, *p* = 0.658) [46]. Liu et al. (2024) studied the effects of chewing gum containing 3 mg/kg of caffeine for 10 min and 15 min before exercise on the stationary free throw shooting test in 15 basketball players. The results of the study showed that caffeinated chewing gum significantly improved stationary free throw shooting test performance (CAF: 79.0 ± 14.3%, PLA: 73.0 ± 9.16%, *p* = 0.012, ES: 0.94) [12].

### 3.11. Heart Rate (HR) and Heart Rate Variability (HRV)

Heart rate variability (HRV) is used as a reference marker to observe the activation of SNS and PNS. Table 7 provides information and outcomes on heart rate (HR) and heart rate variability (HRV). Two studies investigated the effects of caffeinated chewing gum on ANS. A study by Thomas et al. (2017) investigated the effects of different types of genes and caffeinated chewing gum on HRV after exercise. A total of 20 untrained healthy adults chewed gum containing 300 mg of caffeine for 5 min 5 min prior to exercise and HRV was collected after moderate-intensity exercise. The results of the study showed a significant reduction in HRV after moderate-intensity exercise in the A/A homozygotes participant ApEn during 5–10 min post exercise (CAF: 0.8 ± 0.1, PLA: 0.9 ± 0.1, *p* = 0.013, d = 0.96), and in the rate of ApEn recovery (CAF: 0.3 ± 0.2,PLA: 0.4 ± 0.2 ms, *p* = 0.04; d = 0.48) for the caffeinated chewing gum group compared to the placebo group. But CAF did not impair HRV index recovery after exercise [52]. Sargent et al. (2022) found that chewing gum containing 200 mg of caffeine for 5 min before exercise followed by 20 min of moderate-intensity exercise did not delay PNS recovery indexes in HRV (caffeinated chewing gum significantly elevated post-exercise SDNN, LF, SD1, RMSSD) [53]. Most studies have included measures of HR during exercise; however, chewing caffeinated gum before and during exercise did not significantly improve mean HR or peak HR compared to the placebo [13,21,27,32,33,35,37,39,44,50,51,53,54]. Only one study showed that chewing caffeinated gum before exercise significantly increased mid- and post-exercise HR compared with the placebo [40].

### 3.12. Fatigue Indicators

Previous studies have found that caffeine has anti-fatigue effects. This review investigates fourteen studies that included fatigue indicators, such as fatigue index (FI), rating of perceived exertion (RPE), or exercise performance under specific RPE conditions. Table 8 provides information and outcomes on fatigue indicators. Among these, six studies demonstrated that caffeinated chewing gum was effective in reducing RPE or FI. Paton et al. (2010) found that chewing gum containing 240 mg of caffeine for 5 min during repetitive sprint recovery significantly reduced fatigue, and the mean sprint performance power output of well-trained competitive cyclists declined significantly (5.4%, Cohen’s d = 0.25) [43]. Ranchordas et al. (2019) found that chewing gum containing 200 mg of caffeine before exercise significantly reduced fatigue index in 6 × 30 m repetitive sprint performance in competitive university-standard rugby players (CAF: 102.2 ± 0.9; PLA: 103.3 ± 1.2%, *p* = 0.001, ES = 1.03) [35]. Daneshfar et al. (2020) found that pre-exercise chewing of gum containing 300 mg of caffeine significantly reduced RPE in male motocross riders in a bicycle motocross time trial (CAF: 6.6 ± 1.3, PLA: 7.2 ± 1.7, *p* = 0.001, ES = 0.64) [36]. Dittrich et al. (2021) found that chewing gum containing 300 mg of caffeine prior to exercise reduced RPE at exhaustion time in trained endurance runners during 50% maximal aerobic speed TTE performance (CAF: 8 8 ± 2; PLA: 10 4 ± 0.5, *p* < 0.01), but had no significant effect on mean RPE (CAF: 6.2 ± 1.2; PLA: 6.2 ± 1.0, *p* = 0.69) [21]. Liu et al. (2024) found that chewing gum containing 3 mg/kg BW for 10 min and 15 min prior to exercise reduced FI in running-base anaerobic sprint test (RAST) performance in basketball players (CAF: 3.60 ± 1.6%, PLA: 5.21 ± 1.6, *p* = 0.009, ES = 1.00) [12]. Lynn et al. (2024) found that chewing gum containing 300 mg of caffeine for 5 min 30 min prior to exercise reduced the RPE of habitual caffeine-consuming recreational runners in a 5 km parkrun time trial performance (CAF: 15.43, PLA: 16.64, *p* = 0.01) [38]. The results of eight other studies indicated that caffeinated chewing gum did not have a significant difference in RPE during exercise, nor in exercise performance at specific RPE intensities, compared with the placebo group [13,27,32,37,39,48,50,54].

### 3.13. Perceived Pain

Perceived pain can significantly affect an athlete’s performance and overall experience during training and competition. Three studies have included perceived pain as one of their indicators, one of which showed that caffeinated gum significantly reduced perceived pain compared to a placebo. Bellar et al. (2011) found that chewing gum containing 100 mg of caffeine for 5 min prior to exercise reduced forearm muscle pain (CAF: 3.45 ± 2.95, PLA: 4.84 ± 2.92, *p* < 0.001, phi: 0.377) during the grip to exhaustion task compared with the placebo group [48]. In comparison with the placebo group, two other studies found that caffeinated chewing gum did not significantly reduce perceived leg pain during bicycling [39,49].

### 3.14. Biochemical Indicators and Energy Metabolism

Biochemical indicators and energy metabolism are essential for understanding how substances like caffeine affect athletic performance. These indicators provide insights into various physiological processes that occur during exercise, allowing researchers to assess the impact of caffeine on the body. Table 9 provides information and outcomes on biochemical indicators and energy metabolism. Thirteen studies evaluated the effects of caffeinated gum chewing on blood and salivary indicators. The indicators evaluated included blood glucose [28,29,33], blood lactate [21,28,29,33,35,40,44,45,50], serum-free fatty acid [39], testosterone [43,45], cortisol [43,45], catecholamine [39], β-endorphin [27] and caffeine [27,31,32].

Paton et al. (2010) found that chewing gum containing 240 mg of caffeine for 5 min during recovery from repeated sprinting increased salivary testosterone concentration (12%, with a moderate effect size 0.50) and relatively decreased salivary cortisol concentration (21%, with a moderate effect size −0.30) [43]. No significant effect on plasma catecholamine (epinephrine and norepinephrine), serum-free fatty acid, blood glucose, or lactate during submaximal cycling to exhaustion compared to the placebo group when caffeinated chewing gum was consumed at different time points (35/15/5 min before test) was found in the study by Ryan et al. (2012) [39]. In another study also conducted by Ryan et al. (2013), caffeinated chewing gum intake at different time points did not affect β-endorphin during a cycling time trial exercise; moreover, caffeine levels significantly increased during exercise after chewing caffeinated gum for 5 min before exercise. However, the same results were not found at 60 or 120 min before exercise [27]. In a study by Bashafaat et al. (2013), no significant difference was shown in blood glucose or lactate after a 1 km and 4 km cycling time trial while chewing caffeinated gum before and after exercise compared with a placebo group [28]. Lane et al. (2014) found that caffeine concentrations increased significantly during 30 min of chewing compared with the quiet rest and placebo groups, and peaked at the end of time trial performance [32]. Supplementation with 200 mg for female/300 mg for male caffeine-containing chewing gum during exercise elevated lactate concentrations in trained cyclists during the latter part of a bicycle ride compared to the placebo, according to a study by Paton et al. (2015) [40]. Oberlin-Brown et al. (2016) also found that supplementation with a total of 200 mg of caffeine-containing chewing gum during exercise resulted in elevated lactate concentrations during the latter part of a 20 km cycling time trial (ES = 0.90 ± 1.09) compared to the placebo. Similarly, a significant difference was observed for changes in blood glucose concentrations compared to the placebo [33]. Siahpoosh et al. (2016) found that repeated chewing of caffeinated gum before exercise had no significant effect on blood glucose concentration and lactate concentration [29]. Evans et al. (2018) found that chewing gum containing 200 mg of caffeine for 10 min or 5 min prior to elevated exercise (CAF: 11.2 ± 2.3, PLA: 10.3 ± 2.6 mM, *p* = 0.035, ES = 0.36) had no effect after a 40 m maximal shuttle run test, compared to the placebo [44]. A study by Ranchordas et al. (2019) found that chewing caffeinated gum before exercise had no significant difference in post-exercise blood lactate concentration compared to the placebo (CAF: 14.4 ± 3.0; PLA: 13.2 ± 2.5 mmol/L, *p* = 0.075, ES: 0.43) [35]. Russell et al. (2020) found that chewing gum containing 400 mg of caffeine for 5 min during a simulated race was associated with an increase in salivary testosterone concentration during repetitive sprinting (increase 70%, *p* < 0.001) in rugby players, but not in salivary cortisol concentration and blood lactate [45]. Dittrich et al. (2021) found that pre-exercise caffeinated gum chewing was not associated with a significant difference in blood lactate concentration during TTE in endurance sport runners compared to the placebo (CAF: 1.99 ± 0.61; PLA: 1.55 ± 0.88 mmol/L, *p* = 0.08) [21]. In a study by Filip-Stachnik et al. (2021), chewing caffeinated gum before exercise was found to have no significant difference on blood lactate concentration in judo player simulation compared to the placebo [50]. Whalley et al. (2021) compared the effects of different modes of caffeine intake on caffeine metabolism and came to the conclusion that there was no significant difference in post-run urinary caffeine or paraxanthine concentration between the different caffeine intake modes [31].

### 3.15. Risk of Bias

**Figure 3:**
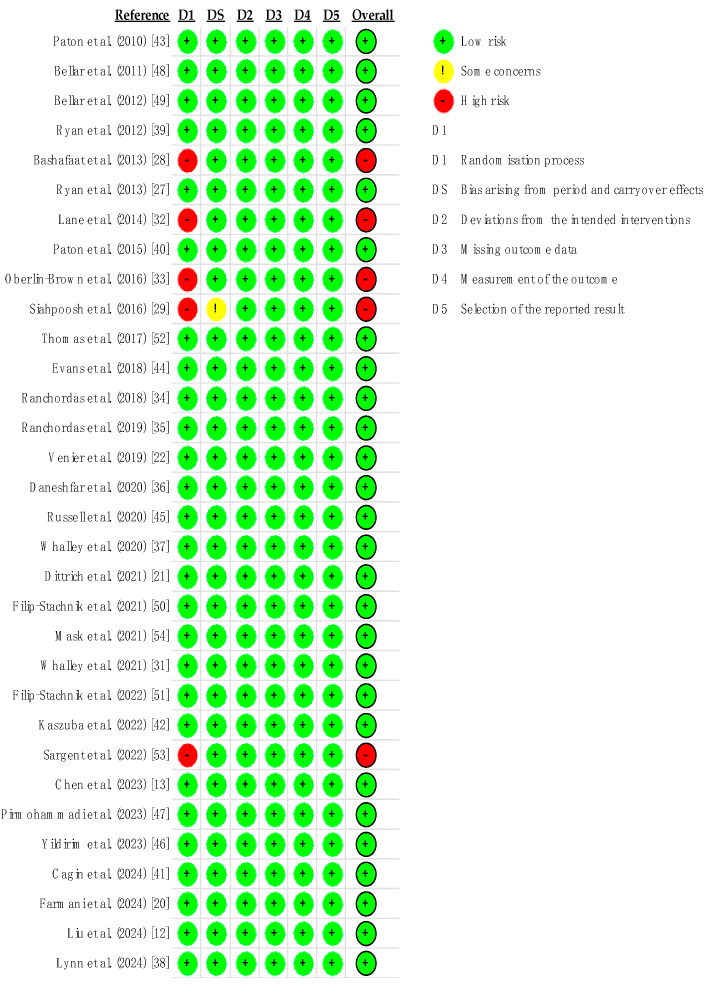
Cochrane Risk-of-Bias (RoB 2) tool.

The present study used the Cochrane risk of bias tool to assess the quality of the included studies’ biases (Figure 3). Five studies did not claim to be randomized controlled trials or to use quasi-experimental design methods, thus presenting a higher risk.

## 4. Discussion

### 4.1. Endurance Exercise Performance

Many studies have demonstrated that pre-exercise caffeine intake enhances endurance exercise performance [4]. Earlier studies suggested that caffeine supplementation may lead to an increase in catecholamine concentrations, the use of FFA as an energy source during exercise, and delayed glycogen depletion [55]. However, in recent years, it has been found that the effects of caffeine on endurance exercise may stem from the effects of caffeine on the central nervous system. When caffeine antagonizes adenosine, it reduces central nervous system fatigue [6]. The effects of caffeine on the periphery, enhancing the release of calcium from the sarcoplasmic reticulum or increasing the activity of the sodium-potassium pump, which enhances muscle contraction, are also important factors in helping caffeine as a stimulus to enhance endurance exercise performance [11]. The results of this systematic review revealed that most studies showed an endurance exercise enhancement effect after chewing caffeinated gum. This suggests that the rapid absorption of caffeine through the oral mucosa allows the body to absorb and utilize the caffeine quickly, which may be effective in enhancing aerobic and endurance exercise performance. However, some studies did not find positive effects of caffeinated chewing gum on endurance exercise, which may be attributed to the type of exercise or the low dose of caffeine. The shorter duration of medium- to long-distance exercise (800 or 1500 m running, 1 or 4 km cycling) may be one of the reasons why caffeinated chewing gum did not enhance performance compared to longer duration endurance exercise (Time to Exhaustion, TTE, or time trial performance) [28]. In addition, lower doses of caffeine than those consumed through chewing gum, although better absorbed and utilized, may not be sufficient to affect energy metabolism, RPE, or pain perception, which in turn may not be able to affect exercise performance [39]. For endurance exercise performance, the relative caffeine concentration in the body may be the main cause of endurance exercise performance.

### 4.2. Sprint Performance

Sprint performance is crucial in team sports. Repetitive short sprints with short recovery times can lead to substrate depletion, metabolite accumulation, and decreased neuromuscular performance [56]. Caffeine supplementation has been shown to be effective in improving sprinting performance and reducing performance decline after repeated sprinting. Compared to repetitive sprinting performance, a single sprinting session is influenced by lower limb strength and explosive power [57,58]. In this systematic review, chewing caffeinated gum was effective in improving basketball players’ 20 m sprint performance [12]. Nonetheless, moderate doses of caffeinated chewing gum intake may not improve short-distance sprint performance in habitual caffeine-using athletes [35,45]. Another study revealed that caffeinated chewing gum had no effects on sprinting and acceleration performance in sprinters, which may be attributed to long-term specialized training, resulting in no beneficial effects of caffeine intake [42].

On the other hand, caffeine intake showed positive benefits on repetitive sprint performance in most studies, which may be related to the resistance to fatigue produced by caffeine intake. Still, in a 2019 meta-analysis, caffeine supplementation did not significantly affect repetitive sprint performance [56]. Compared with previous studies, the minimum dose of caffeine to strengthen repetitive sprint performance must be at least 3 mg/kg [10]. Caffeinated chewing gum intake builds up repetitive sprint performance at lower doses (200–300 mg) of caffeine. Subjects without caffeine intake habits benefited more than subjects with caffeine intake habits [44]. The mode of repeated sprinting measurement, including the sprinting duration and rest periods, also appears to influence caffeine’s benefits [11]. Future studies could examine the effects of caffeinated gum on anaerobic endurance.

### 4.3. Strength Performance

The effects of caffeine on muscle performance include enhancing the ability of the sarcoplasmic reticulum to release calcium, increasing the excitation-contraction coupling of muscle contraction by increasing the activity of the sodium-potassium pump, and promoting muscle performance while decreasing performance decline in muscular endurance exercise [5,8]. For upper-limb strength performance, the articles included in this review consisted of assessments of grip strength and bench press speed. Caffeinated chewing gum helped to improve bench press performance at different speeds. Yet, there was no significant improvement in grip strength. In a review by Grgic (2021), it was found that only caffeine intake of 4–6 mg/kg produced a small (Cohen’s d = 0.07–0.15) gain in upper limb strength [59]. Another meta-analysis showed significant benefits of caffeine supplementation for speed-based resistance training [60]. For lower-limb strength, most studies have found that caffeine supplementation can have a performance-enhancing effect at a lower dose (2 mg/kg) compared with the upper limbs. In the present review, most studies found that caffeinated chewing gum had a performance-enhancing effect on lower limb strength. This result suggests that caffeine is also effective in enhancing lower limb performance through rapid absorption. Some studies have found that the effect of caffeine on lower limb strength may be related to the muscle group, with the knee flexor group showing a less pronounced benefit from caffeine than the knee extensor group [22,46]. Therefore, the effects of caffeine on muscle strength performance may be influenced by motor unit recruitment and muscle activation. Such findings are similar to those of previous reviews [59,61].

In addition, caffeinated chewing gum has been found to have a strength-enhancing effect at relatively low doses compared to traditional caffeine supplementation. Therefore, it is possible that there are other mechanisms besides the traditional ones that influence the effect of caffeinated chewing gum on strength performance. Sympathetic nerve activity, for example, may also play an influential physiological role. By inhibiting adenosine receptors, it increases neuronal excitability and enhances the activity of the nervous system. Increased sympathetic nervous system activity also increases catecholamine level, which increases heart rate, blood pressure, and muscle tone. In addition, the activation of the sympathetic nervous system will lead to vasoconstriction, increasing blood flow to the muscles, which in turn provides more oxygen and nutrients. Therefore, caffeine intake using caffeinated chewing gum may directly or indirectly enhance muscle performance in response to sympathetic activation.

### 4.4. Explosive Power Performance

In this systematic review, two studies that involved tests of upper limb explosive power were included [20,47]. Both studies used medicine ball throwing as an assessment measure and found that caffeinated chewing gum was not effective in enhancing exercise performance. The effect of caffeine on explosive performance was attributed to an increase in calcium flow and an increase in the number of units recruited [11]. In a meta-analysis by Grgic and Varovic (2022), caffeine intake of >3 mg/kg was shown to be effective in improving throwing performance, including throwing speed and distance [62]. In the present studies, however, the reasons for the failure of caffeinated chewing gum to enhance explosive power may be due to the insufficient intake of caffeine, the habit of caffeine use, or the training level of the subjects. In addition, although two studies found that the CAF group outperformed the PLA group, this may not be statistically significant due to sample size [20,47].

CMJ and SJ are important indices for assessing the performance of lower-limb explosive power, where CMJ involves the assessment of rapid force generation during the stretch-shorting cycle and SJ is the ability to respond to muscle contraction during the concentric phase [63]. In a meta-analysis by Grgic et al. (2018), it was shown that caffeine contributes to the enhancement of athletic performance using vertical jump performance as an indicator of lower-limb explosive performance. It is also highly correlated with performance enhancement in many specialized sports, such as basketball and volleyball [64]. In present systematic reviews, caffeine chewing gum was found to have inconsistent effects on lower-limb explosive performance. Some studies have found that lower doses of caffeinated chewing gum can enhance lower-limb explosive and jumping performance. However, some studies failed to find such gains, which may be attributed to the fact that athletes undergoing prolonged training [12,46] or having a caffeine-using habit [20,42] do not benefit from lower doses of caffeine supplementation.

### 4.5. Agility Performance

Change of direction (COD) is one of the most important abilities in team sports (such as soccer, basketball, etc.). Despite the fact that performance of COD may be affected by the ability to sprint and change the speed of movement in sports, fewer studies have found positive results of caffeine on agility performance. In Lazic et al. (2022), a systematic review exploring the effects of caffeine intake on basketball-specific performance, two articles included caffeine intake on agility performance, yet had inconsistent results [65]. Another systematic review by Gomez-Bruton et al. examined the effect of caffeine intake on athletic performance in female team athletes. No significant effects of caffeine intake on agility performance were found [66]. In the other systematic review and meta-analysis by Ferreira et al. (2021), caffeine chewing gum was found to have a beneficial effect on neither agility performance nor cognitive tests [67]. In the present systematic review, caffeinated chewing gum was also found to have inconsistent results on agility performance. Different testing methods and individual sensitivity to caffeine may have contributed to the inconsistent findings [47]. Therefore, whether caffeine chewing gum can improve COD ability or even be applied on the competition field remains to be discussed.

### 4.6. Balance Performance

The effects of caffeine on balance may arise from improved muscle control in the lower limbs and cognitive functions, such as improved attention [68]. The systematic review by Briggs et al. (2021) suggests that caffeine intake’s effect on balance control is affected by age, physical or mental fatigue, and sleep quality. In terms of age, caffeine ingestion did not significantly affect young subjects, but impaired balance was found in older adults [69]. In the present systematic review, after excluding factors like sleep deprivation or sleep disorders, only one article was screened and found that caffeinated chewing gum did not affect balance performance in the younger age group, which is consistent with Briggs’ results. Therefore, the effect of caffeinated chewing gum on homeostasis in different age groups or states could be further investigated in the future.

### 4.7. Cognitive Function and Reaction Performance

Caffeine is the most commonly used central neurostimulant to improve cognitive function performance by enhancing the release of excitatory neurotransmitters, increasing alertness, and reducing the perception of fatigue [70]. A review by Lorenzo et al. (2021) suggests that the effect of caffeine on cognitive function performance may depend on the type of sport that the subject is exercising. Caffeine supplementation would have a more pronounced effect on sports with lower attention requirements than for team athletes who require higher levels of concentration during exercise [70]. In addition, Bellar et al. (2012) found that chewing caffeinated gum enhanced cognitive function and athletic performance in the early morning. Therefore, caffeinated chewing gum may be an effective option for improving cognitive function in the morning [49]. On the other hand, caffeine intake leads to sympathetic activation. Previous studies have shown that low doses of caffeine increase cortical activation and reduce anxiety, whereas high doses increase feelings of nervousness and anxiety [71]. Sympathetic overactivation, along with lower parasympathetic activity, may result in poorer cognitive performance in healthy populations [72]. Cognitive performance at different levels of arousal is also affected by caffeine doses and sympathetic activations. In subjects with a high alert state, higher doses of caffeine may lead to sympathetic over-activation, resulting in a state of over-excitement, which in turn impairs cognitive functioning. On the contrary, in severely fatigued participants, higher doses of caffeine may create an elevated mental state, which in turn may improve cognitive performance [71].

### 4.8. Specialized Sports Performance

In conclusion of the systematic review, caffeine has been shown to have a beneficial effect on some specialized performances, including shot putter throw performance [49], volleyball players’ jump and attack accuracy performance [42,51], table tennis players’ hand movement speed, movement speed, and table tennis related cognitive tests [47], and basketball stationary free throw shooting tests [12]. Some studies have also shown no significant effects of caffeinated chewing gum on specialized sports performance, including soccer players’ ball-kicking speed [46] and the special judo fitness test [50]. Caffeinated chewing gum has been shown to increase the efficiency of caffeine absorption and utilization, and to enhance physical fitness, cognitive function, or motor control, with beneficial effects on sport-specific performance. In the ISSN (2021) point stand on caffeine and athletic performance, it was noted that although caffeine has beneficial effects on many physical properties, the ability to convert to specialized performance is based on each individual’s response to caffeine, and the side effects need to be watched closely. The effects of caffeine on psychosocial performance should also be carefully evaluated during training and competition, regardless of the mode of caffeine use [11].

### 4.9. HR and HRV

Caffeine intake leads to sympathetic activation and increased catecholamine release, leading to cardiac autonomic regulation that results in an accelerated heart rate [30]. Most of the studies included in this systematic review found that HR increased with exercise intensity, but caffeine intake did not induce higher HR compared to the placebo, and only one study found that caffeinated chewing gum group increased HR during the post-exercise phase of exercise compared to the placebo, and in this study, caffeine chewing gum intake also increased post-exercise performance and blood lactate concentration. Therefore, the increase in HR may be attributed to the increase in exercise intensity [40]. The increase in HR during exercise is partly due to the decrease in vagal tone. Therefore, recovery of HR after exercise is a key factor in assessing the reactivation of the vagus nerve and is one of the indicators of mortality risk reduction [73]. HRV, which is composed of changes in continuous heartbeat intervals, is regulated by the ANS, which responds to neurocardiac function and activation of the SNS and PNS. In this literature review, the effect of caffeinated chewing gum on HRV after exercise was included in two articles. The results showed that caffeinated chewing gum did not delay PNS recovery after exercise [51,52]. This result is in line with the meta-analysis of Porto et al. (2022), which showed that caffeine intake did not delay the reactivation of the vagus nerve [30]. In contrast, the meta-analysis of Benjamim et al. (2020) showed that caffeine intake resulted in elevated HR during and after exercise and delayed HR recovery after exercise. Post-exercise recovery of the vagal nerve is influenced by caffeine dose, type of exercise, and the cardiorespiratory fitness of the subject [74]. The two articles included in this study both tested healthy adults at low to moderate doses, and therefore may have contributed to the faster reactivation of the PNS. There is little literature in past research suggesting that the reason caffeine causes elevated exercise performance may come from activation of the sympathetic nerves. Therefore, the rapid caffeine absorption effect of caffeine chewing gum may further enhance sympathetic nerve activity, which may be another effective physiological mechanism for enhancing exercise performance. In order to further validate this mechanism, the effects of caffeine capsules and caffeinated chewing gum on sympathetic nerve activity can be compared at the same concentration of caffeine in the body.

### 4.10. Fatigue-Related Indicators

Decreased RPE during exercises resulting from caffeine intake has been suggested as one of the keys to caffeine’s significant enhancement of exercise performance [61]. In a meta-analysis by Doherty and Smith (2005), it was found that caffeine intake may reduce RPE during exercise, but not in the immediate post-exercise period [75]. Furthermore, in the review by Grgic et al. (2019), it was noted that most RPE for resistance training is measured post-exercise, and that caffeine still has the effect of reducing RPE [61]. In this systematic review, caffeinated chewing gum was found to produce a significant reduction in fatigue index (FI). Such results suggest that caffeinated chewing gum may be effective in mitigating performance decline during the post-exercise period. In addition, caffeine intake had a more pronounced beneficial effect in trained subjects in comparison with untrained subjects [75]. In some studies, caffeinated chewing gum chewing prior to exercise was effective in enhancing exercise performance even though there was no significant reduction in RPE [13,27,31,32]. These results suggest that subjective measures of exercise intensity are only indicative, and that caffeinated chewing gum is still a supplement that can be tried to enhance exercise performance.

### 4.11. Perceived Pain

Muscle pain during exercises negatively affects motor unit recruitment and skeletal muscle strength, which in turn affects performance [76]. Caffeine may reduce muscle pain during exercise by acting on the CNS or PNS. Elevated levels of adenosine in skeletal muscle may result in the transmission of pain signals when it binds to mitochondrial A1 or A2A receptors in nerve endings [77]. Caffeine has an antagonistic effect on adenosine, which in turn reduces pain perception at the central or peripheral level. Caffeine also produces an increase in dopamine concentration in the central nervous system, leading to a decrease in pain perception [11]. In this review, only one study found that caffeinated chewing gum had a positive effect on pain reduction during exercise [48], while two other studies did not show any significant effects [27,39]. Compared with previous studies, Bellar’s study showed that a lower dose of caffeinated chewing gum was associated with a reduction in perceived muscle pain during upper extremity exercise. The fact that the other two studies did not find a positive benefit may be attributed to the exercising of muscles and the lower dose of caffeine. Previous studies have found that higher doses of caffeine (5 mg/kg or 10 mg/kg) can improve pain perception during cycling [78,79,80]. Although caffeine has been shown to have an ameliorative effect on the reduction of pain during and after exercise [81], the inconsistent results of caffeine intake using chewing gum on pain perception remain to be investigated in future studies.

### 4.12. Biochemical Indicators and Energy Metabolism

Previous studies have suggested that the beneficial effects of caffeine on exercise performance may result from central nervous activation, where caffeine binds to adenosine receptors, leading to increased catecholamine concentrations, enhanced FFA oxidation, and delayed glycogen depletion. Such a mechanism was later suggested to be unable to explain the beneficial effects of caffeine during short-term, high-intensity exercise [11]. Assessing blood glucose concentration, serum-free fatty acid, or RER during exercise may help researchers to understand energy metabolism during exercise. Seven studies have evaluated the effects of caffeinated chewing gum chewing on these indices. Only the one by Oberlin-Brown et al. (2016) found that caffeinated chewing gum consumption before and during exercise showed a blood glucose change increase [33]. The remaining six studies concluded that chewing caffeinated gum before and during exercise did not affect blood glucose concentration [28,29,39], serum-free fatty acid [39], or RER [21,27,54] during exercise. Meanwhile, caffeinated chewing gum has been found to have a beneficial effect on exercise performance in some studies [21,27], and the ISSN (2021) position stand suggests that lower doses of caffeine (1–3 mg/kg) intake do not result in altered physiological responses [11]. Also, no changes in EPI and NE concentrations were found with low doses of caffeinated chewing gum in a study by Ryan et al. (2012) [39]. Previous studies have found that increased exercise intensity may result in increased EPI and NE concentrations triggered by sympathetic activation, a response that is mediated by the brainstem and spinal cord [82].

Lactate is often regarded as a metabolic product during exercise and a marker of fat oxidation in skeletal muscle during exercise [83]. Caffeine intake activates sympathetic nerves and stimulates adrenaline secretion, which in turn increases blood lactate concentration during exercise. In addition, the increase in exercise intensity associated with caffeine intake may contribute to the increase in blood lactate concentration. Ten studies included blood lactate concentration as one of the indexes, three of which found that caffeinated chewing gum chewing significantly increased lactate concentration after exercise compared with the placebo. The remaining studies found that caffeinated chewing gum chewing did not significantly increase post-exercise lactate concentration compared to the placebo, although most studies showed an increase in pre- and post-exercise lactate concentration [21,28,29,35,39,45,50]. Paton et al. (2015) found that caffeinated chewing gum chewing resulted in a greater increase in post-exercise blood lactate concentration in men than in women. Besides, there was no significant effect on energy metabolism [40]. Similarly, in two other studies, chewing caffeinated gum was found to have an effect on lactate concentration during post-exercise and enhance post-exercise performance [33,44]. Therefore, the increase in lactate concentration is considered to be a result of the increase in exercise intensity rather than a result of energy metabolism.

Exercise may have physiologic and psychological stress responses. Measurement of salivary cortisol can be used to quantify these stress responses [84]. On the other hand, testosterone concentrations during exercise may be influenced by the type of sport, intensity, athlete training and nutritional status. Higher testosterone concentrations result in more recruitment of motor units and greater force production. Caffeine intake activates the central nervous system and raises testosterone concentrations synergistically, which in turn strengthens exercise performance, as in the findings of studies by Paton et al. (2010) and Russel et al. (2020) [43,45,61]; caffeine chewing gum intake seems to have beneficial effects compared with the increase in cortisol concentration led by exercise. Two studies revealed that chewing caffeinated gum reduced or maintained cortisol concentrations and attenuated the stress response to high-intensity exercise [43,45].

Three studies included indicators of caffeine concentration in the blood. The results showed that chewing caffeinated gum 5–10 min before exercise significantly increased caffeine levels during exercise [27,31,32]. Such results indicate that caffeine chewing gum has the benefits of rapid absorption and utilization. In comparison with capsules, the absorption efficiency of caffeine chewing gum was first investigated in a study by Kamimori et al. (2002) [17]. Subsequently, many studies have started to adapt caffeine chewing gum as the most researched and practically applied method. In addition to absorption through the oral mucosa, caffeine may be absorbed in the intestines along with saliva, with a second peak concentration occurring 40 min after chewing [18]. Therefore, caffeine intake through chewing gum may be a favorable way to utilize caffeine for short periods of time.

### 4.13. Strength and Limitation

This study included 32 research articles, more than half of which were published in the last five years. By evaluating these studies, we gained a comprehensive understanding of the effects of caffeinated chewing gum on exercise performance and related physiological responses. However, several limitations remain in this systematic review. Participants’ caffeine consumption habits may influence their performance after caffeine intake. Although most studies assessed these habits during the screening phase and included participants with low to moderate caffeine consumption levels, the effects of caffeinated chewing gum could still vary among different groups based on their caffeine intake. Additionally, while the rapid absorption and utilization of caffeinated gum offers benefits, whether these effects on exercise performance involve other physiological mechanisms still requires further investigation.

## 5. Conclusions

In many studies of caffeine supplementation on athletic performance with the form of caffeinated chewing gum, it was found to have a positive effect on improving athletic performance. In other studies, some researchers have discovered that caffeine supplementation in the form of caffeinated gum can still be an effective method for enhancing athletic performance but with greater effect sizes and achievable usage of a lower dose caffeine than is typically recommended. Caffeine being rapidly and completely absorbed into the blood within the ingestion of caffeinated chewing gum may result in disparate physiological responses. However, there is still a lack of substantial evidence for these physiological changes to confirm this result, for example, the response to sympathetic nerves. Future studies could pursue the examination on the relationship between caffeinated chewing gum on sympathetic activation and exercise performance.

## Figures and Tables

**Table 1:** Effect of caffeinated chewing gum on endurance exercise performance.

Reference	Participant (Male/Female) Type	CAF Dosage	Chewing Duration	Ingestion Timing (Before Test)	Main Outcome	CAF	PLA	*p*	ES
Ryan et al. (2012) [39]	n = 8 (8/0) College-aged physical active	200 mg	5 min	one of three time points: 35 min; 5 min; 15 min after test	↔ TTE at 85% VO2max			0.980	
Bashafaat et al. (2013) [28]	n = 15 (15/0) Time trial cyclist	180 mg 300 mg	5 min	three time points 180 mg at 30 min 300 mg at 5 min immediately after the 1 and 4 km cycling	↔ 1 km or 4 km time trial			>0.05	
Ryan et al. (2013) [27]	n = 8 (8/0) College-aged trained cyclist	300 mg	5 min	one of three time points: 120 min; 60 min; 5 min	*↓ finish time at trial ingested 5 min before test	38.7 ± 1.2	40.7 ± 1.2	0.023	
Lane et al. (2014) [32]	n = 24 (12/12) Competitive cyclists or triathletes	3 mg/kg	10 min	2 mg/kg: 40 min 1 mg/kg: 10 min	*↑ Mean power output (W)	260 ± 58	250 ± 57	<0.001	
					*↓ Time trial finish time	↑ 1.3% for male ↑ 1.6% for female		<0.001	
Paton et al. (2015) [40]	n = 20 (10/10) trained cyclist	male: 300 mg female: 200 mg	5 min	immediately following the first maximal sprint at 10 km	↔ first 20 km performance or physiological				
					*↑ mean power output during the third lap				
Oberlin-Brown et al. (2016) [33]	n = 11 (11/0) Endurance-trained cyclists or triathletes	4 × 50 mg	5 min	every 25% time trial	↔ 20 km time trial (m:s)	32:20 ± 1:57	32:27 ± 1:57		
					↔ Mean power output (W)	273 ± 40	270 ± 37		0.03
					*↑ Mean power output for 10–15 km (W)	263 ± 39	259 ± 35		0.09
					*↑ Mean power output for 15–20 km (W)	284 ± 42	273 ± 41		0.24
Siahpoosh et al. (2016) [29]	n = 10 (10/0) Middle-distance runners	180 mg 300 mg	5 min	three time point: 30 min; 5 min immediately after test	↔ 800 m running	2.03 ± 0.41 (180) 2.02 ± 0.29 (300)	2.04 ± 0.43	0.095	
					↔ 1500 m running	4.17 ± 0.18 (180) 4.18 ± 0.36 (300)	4.22 ± 0.23	0.108	
Ranchordas et al. (2018) [34]	n = 10 (10/0) University-standard soccer players	200 mg	5 min	5 min	*↑ Covered distance in YO-YO IR1 test	1754 ± 156	1719 ± 139	0.016	0.24
Ranchordas et al. (2019) [35]	n = 17 (17/0) competitive university-standard rugby players	200 mg	5 min	immediately	*↑ Covered distance in YO-YO IR2 test	426 ± 105	372 ± 91	0.010	0.55
Daneshfar et al. (2020) [36]	n = 14 (14/0) male motocross riders	300 mg	NA	NA	*↑ Time trial performance			0.001	0.71
					*↑ Peak power to weight ratio			0.001	0.79
					*↑ Maximal power to weight ratio			0.001	0.80
Whalley et al. (2020) [37]	n = 14 amateur runner	NA	NA	15 min	*↑ 5 km running time trial	↑ 0.9% ± 1.4%		<0.005	
Dittrich et al. (2021) [21]	n = 12 (12/0) trained endurance runners	300 mg	5 min	NA	*↑ TTE at 50% MAS (min)	40.60 ± 8.53	33.23 ± 7.41	<0.001	
					*↑ Total distance coverd (km)	10.36 ± 2.19	8.45 ± 1.73	<0.001	
Whalley et al. (2021) [31]	n = 14 (10/4) experienced runners	65 kg↓: 200 mg 65 kg↑: 300 mg	NA	15 min	↔ 5 km runnung time trial				
Farmani et al. (2024) [20]	n = 18 (18/0) Table tennis player	65 kg↓: 200 mg 65 kg↑: 300 mg	10 min	immediately	*↑ TTE (min)	12.26 ± 1.30	11.58 ± 1.22	<0.001	
					*↑ VO2 at VT1 (L/min)	25.89 ± 2.27	24.26 ± 1.57	0.004	
					*↑ VO2 at RCP (L/min)	31.35 ± 2.92	27.93 ± 2.64	<0.001	
					↔ VO2max (ml/min/kg)	50.11 ± 5.14	50.50 ± 5.98	0.877	
Lynn et al. (2024) [38]	n = 36 (31/5) Recreational runner	300 mg	5 min	30 min	*↓ 5 km parkrun time trial	↓ 17.28 s		0.01	
					↔ Split time			0.06	
					↔ Pacing			0.21	

Data are reported as mean ± SD. CAF caffeinated chewing gum trial, PLA placebo trial, ES effect size, *↑ Significantly greater than PLA, *↓ Significantly less than PLA, ↔ No different between trials, TTE time to exhaustion, W watt, IR intermittent recovery, VT1 ventilatory threshold, RCP respiratory compensation point, ↑ improve performance, ↓ decreased.

**Table 2:** Effect of caffeinated chewing gum on sprint performance.

Reference	Participant (Male/Female) Type	CAF Dosage	Chewing Duration	Ingestion Timing (Before Test)	Main Outcome	CAF	PLA	*p*	ES
Paton et al. (2010) [43]	n = 9 (9/0) Well-train competitive cyclist	240 mg	5 min	during a 10 min recovery period after completion of the second exercise set.	*↓ Mean power output declined	0.4 ± 7.7%	5.8 ± 4.0%		0.25 ± 0.16
Paton et al. (2015) [40]	n = 20 (10/10) trained cyclist	male: 300 mg female: 200 mg	5 min	immediately following the first maximal sprint at 10 km	*↑ sprint power output during the third lap				
		
Evans et al. (2016) [44]	n = 18 (18/0) Team sport athletes	200 mg	10 min	5 min	↔ 40 m MST total time (s)	87.5 ± 3.1	87.8 ± 3.0	0.214	0.10
					↔ 40 m MST fastest sprint time (s)	8.33 ± 0.23	8.33 ± 0.20	0.879	0.02
					↔ 40 m MST slowest sprint time (s)	9.06 ± 0.39	9.10 ± 0.43	0.452	0.10
					↔ Overall sprint performance decrement (%)	5.00 ± 2.84	5.43 ± 2.68	0.209	0.16
					*↓ Sprint performance decrement for low habitual participants (%)	5.33 ± 3.12	6.53 ± 2.91	0.049	0.33
					↔ Sprint performance decrement for moderate-to-high habitual participants (%)	3.98 ± 2.57	3.80 ± 1.79	0.684	0.08
Ranchordas et al. (2018) [34]	n = 10 (10/0) University-standard soccer players	200 mg	5 min	5 min	↔ 20 m sprint test (s)	3.2 ± 0.3	3.1 ± 0.3	0.567	0.33
Ranchordas et al. (2019) [35]	n = 17 (17/0) competitive university-standard rugby players	200 mg	5 min	immediately	↔ 6 × 30 m RSP			0.341	
Russell et al. (2020) [45]	n = 14 (14/0) Professional academy rugby player	400 mg	5 min	10 min	↔ RSP				
Kaszuba et al. (2022) [42]	n = 12 (9/3) volleyball players	male: 300 mg female: 200 mg	5 min	15 min	↔ 5 m sprint (s)	0.95 ± 0.11	0.95 ± 0.11	1.000	
					↔ 10 m sprint (s)	1.69 ± 0.12	1.68 ± 0.13	0.619	
Cagin et al. (2024) [41]	n = 31 (13/18) sprinters	5 mg/kg	NA	15 min	*↓ 30 m sprint reaction time (s)	0.17 ± 0.22	0.19 ± 0.02	0.020	
					↔ 30 m sprint acceleration (s)	3.36 ± 0.06	2.89 ± 0.05	0.690	
					↔ 30 m sprint total time (s)	5.83 ± 0.09	5.06 ± 0.09	0.43	
Liu et al. (2024) [12]	n = 15 (15/0) basketball player	3 mg/kg	10 min	15 min	*↑ 20 m sprint split performance (0–10 m)			0.045	0.94
					*↑ 20 m sprint split performance (10–20 m)			0.019	0.70
					↔ RAST peak power (W)	1354.86 ± 44.2	1326.70 ± 75.0	0.328	
					*↑ RAST mini power (W)	1234.44 ± 75.7	1153.90 ± 35.9	0.008	1.35
					↔ RAST peak power per weight (W)	17.92 ± 1.9	17.53 ± 1.8	0.323	
					*↑ RAST mini power per weight (W)	16.30 ± 2.1	15.26 ± 1.6	0.011	0.53

Data are reported as mean ± SD. CAF caffeinated chewing gum trial, PLA placebo trial, ES effect size, *↑ Significantly greater than PLA, *↓ Significantly less than PLA, ↔ No different between trials. MST maximal shuttle run test, RSP Repeated sprint performance, RAST running-based anaerobic sprint test, W watt.

**Table 3:** Effect of caffeinated chewing gum on strength performance.

Reference	Participant (Male/Female) Type	CAF Dosage	Chewing Duration	Ingestion Timing (Before Test)	Main Outcome	CAF	PLA	*p*	ES
Bellar et al. (2011) [48]	n = 10 (5/5) Healthy college age	100 mg	5 min	immediately	↔ Grip to exhaustion (s)	104.98 ± 57.95	99.85 ± 78.39	0.786	0.009
Venier et al. (2019) [22]	n = 19 (19/0) Healthy adult	300 mg	10 min	immediately	*↑ Peak torque at angular velocity of 60°/s for knee extensor (Nm)	245.0 ± 43.3	236.6 ± 36.2	0.048	0.21
					*↑ Average power at angular velocity of 60°/s for knee extensor (W)	180.0 ± 34.1	172.1 ± 29.1	0.031	0.25
					*↑ Peak torque at angular velocity of 60°/s for knee flexion (Nm)	142.7 ± 25.5	137.1 ± 25.4	0.040	0.22
					↔ Average power at angular velocity of 60°/s for knee flexion (W)	111.3 ± 21.3	109.5 ± 21.4	0.320	0.09
					↔ Peak torque at angular velocity of 180°/s for knee extensor (Nm)	170.2 ± 28.7	164.4 ± 23.8	0.073	0.22
					*↑ Average power at angular velocity of 180°/s for knee extensor (W)	322.1 ± 59.1	306.2 ± 48.4	0.035	0.30
					*↑ Peak torque at angular velocity of 180°/s for knee flexion (Nm)	107.6 ± 17.6	101.7 ± 19.9	0.021	0.31
					↔ Average power at angular velocity of 180°/s for knee flexion (W)	196.6 ± 41.9	188.8 ± 48.9	0.265	0.17
					*↑ Average power at angular velocity of 180°/s for knee extensor (W)				
					*↑ Bench press velocity at 50% 1 RM (m/s)	0.85 ± 0.08	0.82 ± 0.09	0.044	0.30
					*↑ Bench press velocity at 75% 1 RM (m/s)	0.57 ± 0.07	0.54 ± 0.06	0.005	0.44
					*↑ Bench press velocity at 90% 1 RM (m/s)	0.38 ± 0.07	0.35 ± 0.07	0.002	0.43
					*↑ Peak power output on the rowing ergometer test (W)	667.5 ± 78.5	635.9 ± 68.7	0.006	0.41
Dittrich et al. (2021) [21]	n = 12 (12/0) trained endurance runners	300 mg	5 min	NA	*↓ Reduction inf MVC after exercise (N/m)	pre: 279.9 ± 56.5; post: 244.0 ± 46.3	pre: 280.4 ± 52.8; post: 237.7 ± 50.5		
					↔ Muscle activity				
Chen et al. (2023) [13]	n = 19(19/0) Healthy adult	200 mg	5 min	10 min	*↑ Flywheel RDL peak concentric power (W)			0.016	0.44
					*↑ Flywheel RDL peak eccentric power (W)			0.005	0.55
					*↑ Flywheel RDL average power (W)			0.013	0.43
					↔ Flywheel RDL average force			0.063	0.50
					*↑ Flywheel RDL total work (W)			0.026	0.28
Pirmohammadi et al. (2023) [47]	n = 18 (0/18) table tennis player	65 kg↓: 200 mg 65 kg↑: 300 mg	10 min	immediately	↔ Handgrip strength (kg)	55.27 ± 8.30	53.05 ± 7.5	0.311	
Yildirim et al. (2023) [46]	n = 14 (14/0) highly trained soccer player	100 mg 200 mg	5 min	15 min	*↑ quadriceps strength (kg)	200 mg: 53.77 ± 5.77 100 mg: 49.62 ± 8.81	49.20 ± 7.20	M > L 0.048	0.55
								M > P 0.032	0.70
					↔ Hamstring strength (kg)	200 mg: 28.11 ± 6.12 100 mg: 26.81 ± 5.83	25.66 ± 3.49	0.251	
					↔ Handgrip strength (kg)	200 mg: 48.76 ± 6.53 100 mg: 48.16 ± 6.22	47.07 ± 6.82	0.145	
Liu et al. (2024) [12]	n = 15 (15/0) basketball player	3 mg/kg	10 min	15 min	*↑ Average power for flywheel squat (W)			0.012	0.41
					*↑ Peak concentric power for flywheel squat (W)			0.013	0.48
					*↑ Peak eccentric power for flywheel squat (W)			0.028	0.45

Data are reported as mean ± SD. CAF caffeinated chewing gum trial, PLA placebo trial, ES effect size, *↑ Significantly greater than PLA, *↓ Significantly less than PLA, ↔ No different between trials RDL Romanian deadlift, W watt, ↑ more than, ↓ less than.

**Table 4:** Effect of caffeinated chewing gum on explosive power performance.

Reference	Participant (Male/Female) Type	CAF Dosage	Chewing Duration	Ingestion Timing (Before Test)	Main Outcome	CAF	PLA	*p*	ES
Ranchordas et al. (2018) [34]	n = 10 (10/0) university-standard soccer players	200 mg	5 min	5 min	*↑ CMJ (cm)	47.1 ± 3.4	46.1 ± 3.2	0.008	0.30
Ranchordas et al. (2019) [35]	n = 17 (17/0) competitive university-standard rugby players	200 mg	5 min	immediately	*↑ CMJ (cm)	43.7 ± 7.6	42.2 ± 6.2	0.044	0.22
Kaszuba et al. (2022) [42]	n = 12 (9/3) volleyball players	male: 300 mg female: 200 mg	5 min	15 min	↔ CMJ (cm)	51.2 ± 11.2	51.0 ± 11.4	0.820	
					↔ SJ (cm)	39.1 ± 7.8	40.9 ± 9.6	0.230	
Pirmohammadi et al. (2023) [47]	n = 18 (0/18) table tennis player	65 kg↓: 200 mg 65 kg↑: 300 mg	10 min	immediately	*↑ Sargent’s jump test (Watt)	1865.11 ± 452.00	1689.55 ± 49.68	0.001	
					↔ throwing medicine ball (cm)	448.33 ± 61.59	438.66 ± 44.76	0.192	
Yildirim et al. (2023) [46]	n = 14 (14/0) highly trained soccer player	100 mg 200 mg	5 min	15 min	↔ Peak jump heights (cm)	200 mg: 34.62 ± 5.42 100 mg: 37.48 ± 7.39	37.27 ± 4.57	0.678	
					↔ Mean jump heights (cm)	200 mg: 30.20 ± 6.31 100 mg: 32.23 ± 5.55	32.43 ± 3.95	0.833	
					↔ Peak jump power (Watt/kg)	200 mg: 43.70 ± 6.00 100 mg: 46.12 ± 7.85	45.91 ± 6.26	0.377	
					↔ Mean jump power (Watt/kg)	200 mg: 40.04 ± 6.05 100 mg: 41.69 ± 5.74	41.86 ± 5.01	0.439	
Farmani et al. (2024) [20]	n = 18 (18/0) table tennis player	65 kg↓: 200 mg 65 kg↑: 300 mg	10 min	immediately	↔ throwing medicine ball	6.87 ± 0.92	6.57 ± 0.97	0.928	
					↔ Sarjent’s jump height (cm)	43.00 ± 5.32	42.00 ± 5.79	0.596	
Liu et al. (2024) [12]	n = 15 (15/0) basketball player	3 mg/kg	10 min	15 min	↔ CMJ (cm)			0.147	

Data are reported as mean ± SD. CAF caffeinated chewing gum trial, PLA placebo trial, ES effect size, *↑ Significantly greater than PLA, ↔ No different between trials, CMJ countermovement jump, SJ squat jump, ↑ more than, ↓ less than.

**Table 5:** Effect of caffeinated chewing gum on agility performance.

Reference	Participant (Male/Female) Type	CAF Dosage	Chewing Duration	Ingestion Timing (Before Test)	Main Outcome	CAF	PLA	*p*	ES
Ranchordas et al. (2019) [35]	n = 17 (17/0) competitive university-standard rugby players	200 mg	5 min	immediately	↔ Illinois agility test (s)	16.22 ± 1.08	15.88 ± 1.09	0.271	
Kaszuba et al. (2022) [42]	n = 12 (9/3) volleyball players	male: 300 mg female: 200 mg	5 min	15 min	↔ Modified agility T-test (s)	9.44 ± 0.69	9.45 ± 0.77	0.952	
Pirmohammadi et al. (2023) [47]	n = 18 (0/18) table tennis player	65 kg↓: 200 mg 65 kg↑: 300 mg	10 min	immediately	*↑ Edgren’s agility test (score)	24.38 ± 2.19	23.22 ± 2.41	0.002	
Liu et al. (2024) [12]	n = 15 (15/0) basketball player	3 mg/kg	10 min	15 min	↔ Agility T-test			0.571	

Data are reported as mean ± SD. CAF caffeinated chewing gum trial, PLA placebo trial, ES effect size, *↑ Significantly greater than PLA, ↔ No different between trials, ↑ more than, ↓ less than.

**Table 6:** Effect of caffeinated chewing gum on specialized sports performance.

Reference	Participant (Male/Female) Type	CAF Dosage	Chewing Duration	Ingestion Timing (Before Test)	Main Outcome	CAF	PLA	*p*	ES
Bellar et al. (2012) [49]	n = 9 (4/5) college shot putter	100 mg	5 min	immediately	*↑ first throw test performance (m)	9.62 ± 1.71	9.05 ± 1.69	0.050	0.99
					*↑ first group of three throws			0.067	0.35
Filip-Stachnik et al. (2021) [50]	n = 9 (9/0) healthy experienced judoists	200 mg 400 mg	5 min	15 min	↔ Total number of SJFT throws (number)	200 mg 62.22 ± 4.32 400 mg 60.22 ± 4.08	59.66 ± 4.15	0.063	
					↔ SJFT Index			0.099	
Filip-Stachnik et al. (2022) [51]	n = 12 (0/12) volleyball players	400 mg	5 min	15 min	*↑ Attack jump height (cm)	pre-game			0.15
						47.2 ± 7.3	46.0 ± 7.9	0.032	
						post-game			
						47.5 ± 7.5	46.3 ± 8.3	0.022	
					↔ Block jump heigh (cm)	pre-game		0.724	
						33.0 ± 4.5	32.6 ± 5.7		
						post-game			
						34.7 ± 6.2	34.8 ± 6.4		
					↔ Number of jumps during the game (jumps)	52 ± 13	47 ± 15	0.273	

					↔ Volleyball specific skill during the gmae (total points, total errors, service points, service errors, reception errors, negative reception, positive reception, perfect reception, and blocking points, *p* > 0.05)				
Kaszuba et al. (2022) [42]	n = 12 (9/3) volleyball players	male: 300 mg female: 200 mg	5 min	15 min	↔ Attack jump (cm)	61.4 ± 14.9	62.4 ± 13.9	0.342	
					↔ Block jump (cm)	48.4 ± 10.6	48.4 ± 11.6	0.995	
					↔ Standing attack speed (km/h)	82 ± 11	79 ± 12	0.274	
					↔ Attack speed (km/h)	85 ± 14	81 ± 13	0.119	
					↔ Service speed (km/h)	88 ± 14	86 ± 13	0.254	
					*↑ Attack accuracy (points)	18 ± 3	15 ± 4	0.023	0.85
					↔ Serve accuracy (points)	12 ± 4	10 ± 3	0.140	
Pirmohammadi et al. (2023) [47]	n = 18 (0/18) table tennis player	65 kg↓: 200 mg 65 kg↑: 300 mg	10 min	immediately	*↑ hand movement speed (n)	11.08 ± 1.27	12.19 ± 1.43	<0.001	
					*↑ movement speed (s)	3.74 ± 0.22	4.16 ± 4.0	0.001	
					↔ Accuracy in eye-hand coordination test (n)	28 ± 1.45	26.88 ± 2.21	0.091	
					*↑ Cognitive test (n)	22.44 ± 1.5	2.11 ± 1.84	<0.001	
					↔ Accuracy of service (score)	38.11 ± 4.82	36.33 ± 4.02	0.211	
					↔ Counter performance (n)	26.66 ± 1.87	26.83 ± 1.33	0.710	
					↔ Accuracy of forehand drive (score)	16.11 ± 2.58	15.72 ± 1.99	1.000	
					↔ Backhand-push performance (score)	28.05 ± 1.25	28.30 ± 0.94	0.318	
Yildirim et al. (2023) [46]	n = 14 (14/0) highly trained soccer player	100 mg 200 mg	5 min	15 min	↔ Ball-kicking speed (km/h)	200 mg: 106.36 ± 5.79 100 mg: 107.80 ± 4.34	106.74 ± 7.69	0.658	
Liu et al. (2024) [12]	n = 15 (15/0) basketball player	3 mg/kg	10 min	15 min	*↑ stationary free throw shooting test (%)	79.0 ± 14.3	73.0 ± 9.16	0.012	0.94

Data are reported as mean ± SD. CAF caffeinated chewing gum trial, PLA placebo trial, ES effect size, *↑ Significantly greater than PLA, ↔ No different between trials, ↑ more than, ↓ less than.

**Table 7:** Effect of caffeinated chewing gum on heart rate (HR) and heart rate variability (HRV).

Reference	Participant (Male/Female) Type	CAF Dosage	Chewing Duration	Ingestion Timing (Before Test)	Main Outcome	CAF	PLA	*p*	ES
Ryan et al. (2012) [39]	n = 8 (8/0) College-aged physical active	200 mg	5 min	one of three time points: 35 min; 5 min; 15 min after test	↔ HR for TTE at 85% VO2max				
Ryan et al. (2013) [27]	n = 8 (8/0) College-Aged trained cyclist	300 mg	5 min	one of three time points: 120 min; 60 min; 5 min	↔ HR for cycling time trial				
Lane et al. (2014) [32]	n = 24 (12/12) competitive cyclists or triathletes	3 mg/kg	10 min	2 mg/kg: 40 min 1 mg/kg: 10 min	↔ HR for cycling time trial	male:172 ± 10 female: 174 ± 9	male: 167 ± 11 female: 171 ± 8		
Paton et al. (2015) [40]	n = 20 (10/10) trained cyclist	male: 300 mg female: 200 mg	5 min	immediately following the first maximal sprint at 10 km	*↑ HR during the third lap				
Oberlin-Brown et al. (2016) [33]	n = 11 (11/0) endurance-trained cyclists or triathletes	4 × 50 mg (2.7 mg/kg)	5 min	every 25% time trial	↔ Average HR	176 ± 11	163 ± 13		
					↔ Maximal HR	176 ± 10	164 ± 11		
Thomas et al. (2017) [52]	n = 20 (13/7) untrained healthy adults A/A homozygotes n = 11 (7/4) C allele carrier n = 9 (6/3)	300 mg	5 min	5 min	*↓ ApEn for the A/A group during 5–10 min post exercise	0.8 ± 0.1	0.9 ± 0.1	0.013	0.96
					*↓ The rate of ApEn recovery for A/A group	0.3 ± 0.2	0.4 ± 0.2	0.04	0.48
Evans et al. (2018) [44]	n = 18 (18/0) team sport athletes	200 mg (2.5 ± 0.2 mg/kg)	10 min	5 min	↔ HR for 40 m MST			0.366	
Ranchordas et al. (2019) [35]	n = 17 (17/0) competitive university-standard rugby players	200 mg (2.3 ± 0.2 mg/kg)	5 min	immediately	↔ HR for YO-YO IR2 test (beat/min)	173 ± 7	169 ± 14	0.204	0.26
Whalley et al. (2020) [37]	n = 14 amateur runner	3–4.5 mg/kg	NA	15 min	↔ HR for 5 km running time trial				
Dittrich et al. (2021) [21]	n = 12 (12/0) trained endurance runners	300 mg	5 min	NA	↔ HR for TTE at 50% MAS				
Filip-Stachnik et al. (2021) [50]	n = 9 (9/0) healthy experienced judoists	200 mg 400 mg	5 min	15 min	↔HR for SJFT			0.525	
Mask et al. (2021) [54]	n = 16 (0/16) recreationally active college females	300 mg	NA	15 min	↔ HR for Arm ergometer cadence for RPE production trial				
Filip-Stachnik et al. (2022) [51]	n = 12 (0/12) volleyball players	400 mg	5 min	15 min	↔ Mean HR during volleyball game (bpm)	136 ± 10	134 ± 12	0.724	
					↔ Peak HR during vollytball game (bpm)	176 ± 10	178 ± 11	0.794	
Sargent et al. (2022) [53]	n = 18 (9/9) healthy, college-Aged, physically active adults	200 mg	5 min	5 min	*↑ post-exercise SDNN (ms)			0.013	
					*↑ post-exercise LF (m/s^2^)			0.015	
					*↑ post-exercise HF			0.001	
					*↑ post-exercise SD1			0.017	
					*↑ post-exercise RMSSD			0.066	
Chen et al. (2023) [13]	n = 19 (19/0) healthy adult	200 mg	5 min	10 min	↔ HR during RDL on flywheel (bpm)	132.9 ± 18.7	127.9 ± 12.7	0.143	

Data are reported as mean ± SD. CAF caffeinated chewing gum trial, PLA placebo trial, ES effect size, *↑ Significantly greater than PLA, *↓ Significantly less than PLA, ↔ No different between trials.

**Table 8:** Effect of caffeinated chewing gum on fatigue indicators.

Reference	Participant (Male/Female) Type	CAF Dosage	Chewing Duration	Ingestion Timing (Before Test)	Main Outcome	CAF	PLA	*p*	ES
Paton et al. (2010) [43]	n = 9 (9/0) Well-trained competitive cyclist	240 mg	5 min	during a 10 min recovery period after completion of the second exercise set.	*↓ fatigue for RSP	↓ 5.4%			0.25 ± 0.16
Bellar et al. (2011) [48]	n = 10 (5/5) Healthy college age	100 mg	5 min	immediately	↔ RPE for grip to exhaustion	13.45 ± 3.23	13.32 ± 4.15	0.411	
Ryan et al. (2012) [39]	n = 8 (8/0) College-aged physical active	200 mg	5 min	one of three time points: 35 min 5 min 15 min	↔ RPE for time to exhaustion at 85% VO2max				
Ryan et al. (2013) [27]	n = 8 (8/0) College-aged trained cyclist	300 mg	5 min	one of three time points: 120 min 60 min 5 min	↔ RPE for cycling time trial				
Lane et al. (2014) [32]	n = 24 (12/12) competitive cyclists or triathletes	3 mg/kg	10 min	2 mg/kg: 40 min 1 mg/kg: 10 min	↔ RPE for cycling time trial	male: 17 ± 0.8 female: 17 ± 1.0	male: 17 ± 0.8 female: 17 ± 1.2		
Ranchordas et al. (2019) [35]	n = 17 (17/0) competitive university-standard rugby players	200 mg	5 min	immediately	*↓ Fatigue index for RSP (%)	102.2 ± 0.9	103.3 ± 1.2	0.001	1.03
Daneshfar et al. (2020) [36]	n = 14 (14/0) male motocross riders	300 mg	NA	NA	*↓ RPE for bicycle motor cross time trial	6.6 ± 1.3	7.2 ± 1.7	0.001	0.64
Whalley et al. (2020) [37]	n = 14 amateur runner	3–4.5 mg/kg	NA	15 min	↔ RPE for 5 km running time trial				
Dittrich et al. (2021) [21]	n = 12 (12/0) trained endurance runners	300 mg	5 min	NA	*↓ RPE for TTE at 50%MAS at exhaustion time	8.8 ± 1.2	10.4 ± 0.5	<0.01	
Mask et al. (2021) [54]	n = 16 (0/16) recreationally active college females	300 mg	NA	15 min	↔ CAD at RPE 4 (rev/min)	37.7 ± 1.6	37.6 ± 1.6	>0.05	
					↔ CAD at RPE 7 (rev/min)	42.9 ± 1.6	41.2 ± 1.7	>0.05	
Filip-Stachnik et al. (2021) [50]	n = 9 (9/0) healthy experienced judoists	200 mg 400 mg	5 min	15 min	↔ RPE for SJFT			0.538	
Chen et al. (2023) [13]	n = 19(19/0) healthy adult	200 mg	5 min	10 min	↔ RPE for RDL on flywheel	11.7 ± 2.4	12.0 ± 2.5	0.266	
Liu et al. (2024) [12]	n = 15 (15/0) basketball player	3 mg/kg	10 min	15 min	*↓ Fatigie index for RAST (%)	3.60 ± 1.6	5.21 ± 1.6	0.009	1.00
Lynn et al. (2024) [38]	n = 36 (31/5) recreational runner	300 mg	5 min	30 min	*↓ RPE for 5 km parkrun	15.43	16.64	0.01	

Data are reported as mean ± SD. CAF caffeinated chewing gum trial, PLA placebo trial, ES effect size, *↓ Significantly less than PLA, ↔ No different between trials.

**Table 9:** Effect of caffeinated chewing gum on biochemical and energy metabolism indicators.

Reference	Participant (Male/Female) Type	CAF Dosage	Chewing Duration	Ingestion Timing (Before Test)	Main Outcome	CAF	PLA	*p*	ES
Paton et al. (2010) [43]	n = 9 (9/0) Well-trained competitive cyclist	240 mg	5 min	during a 10 min recovery period after completion of the second exercise set.	*↑ Salivary testosterone concentration	↑ 12%			0.50
					*↓ Salivary cortisol concentration	↓ 21%			−0.30
Ryan et al. (2012) [39]	n = 8 (8/0) College-aged physical active	200 mg	5 min	one of three time points: 35 min 5 min 15 min after test	↔ serum-free fatty acid during time to exhaustion at 85% VO2max				
					↔ Plasma epinephrine concentration				
					↔ Plasma norepinephrine concentration				
					↔ Blood glucose				
					↔ Blood lactate				
Bashafaat et al. (2013) [28]	n = 15 (15/0) time trial cyclist	180 mg 300 mg	5 min	three time points: 180 mg at 30 min 300 mg at 5 min immediately after the 1 and 4 km cycling	↔ Blood glucose during time trial for 1 km or 4 km				
					↔ Blood lactate during time trial for 1 km or 4 km				
Ryan et al. (2013) [27]	n = 8 (8/0) College-Aged trained cyclist	300 mg	5 min	one of three time points: 120 min 60 min 5 min	↔ β-endorphin during the cycling time trial				
					*↑ Plasma caffeine concentration during the cycling time trial at trial ingested 5 min before test not in −120 or −60 trial			0.046	
Lane et al. (2014) [32]	n = 24 (12/12) competitive cyclists or triathletes	3 mg/kg	10 min	2 mg/kg: 40 min 1 mg/kg: 10 min	*↑ Blood caffeine concentration within 30 min of ingestion (µmol/L)	10.0 ± 3.80			
					*↑ Peak caffeine concentration (µmol/L)	17.2 ± 5.5			
Paton et al. (2015) [40]	n = 20 (10/10) trained cyclist	male: 300 mg female: 200 mg	5 min	immediately following the first maximal sprint at 10 km	*↑ Blood lactate during the third lap (mmol/L)	5.2 ± 2.3	4.7 ± 2.2		~0.2
Oberlin-Brown et al. (2016) [33]	n = 11 (11/0) endurance-trained cyclists or triathletes	4 × 50 mg (2.7 mg/kg)	5 min	every 25% time trial	*↑ Blood glucose change				−0.44 ± 0.31
					*↑ Blood lactate during post-20 km time trial				0.90 ± 1.09
Siahpoosh et al. (2016) [29]	n = 10 (10/0) middle-distance runners	180 mg 300 mg	5 min	30 min 5 min immediately after test	↔ Blood lactate				
					↔ Blood glucose				
Evans et al. (2018) [44]	n = 18 (18/0) team sport athletes	200 mg (2.5 ± 0.2 mg/kg)	10 min	5 min	*↑ Blood lactate after MST (mM)	11.2 ± 2.3	10.3 ± 2.6	0.035	0.36
Ranchordas et al. (2019) [35]	n = 17 (17/0) competitive university-standard rugby players	200 mg (2.3 ± 0.2 mg/kg)	5 min	immediately	↔ Blood lactate (mmol/L)	14.4 ± 3.0	13.2 ± 2.5	0.075	0.43
Russell et al. (2020) [45]	n = 14 (14/0) professional academy rugby player	400 mg (4.1 ± 0.5 mg/kg)	5 min	10 min	↔ Blood lactate				
					*↑ Salivary testosterone before second RSSA	increase 70%		<0.001	
					↔ Salivary cortisol concerntration			0.307	
Dittrich et al. (2021) [21]	n = 12 (12/0) trained endurance runners	300 mg	5 min	NA	↔ Delta blood lactate before and after TTE at 50% MAS (mmol/L)	1.99 ± 0.61	1.55 ± 0.88	0.080	
Filip-Stachnik et al. (2021) [50]	n = 9 (9/0) healthy experienced judoists	200 mg 400 mg	5 min	15 min	↔ Blood lactate during the SJFT			0.098	
Whalley et al. (2021) [31]	n = 14 (10/4) experienced runners	65 kg↓: 200 mg 65 kg↑: 300 mg	NA	15 min	↔ Post-run urinary caffeine or paraxanthine concentration between different caffeine intake (Gum, Strip, Tablet)				

Data are reported as mean ± SD. CAF caffeinated chewing gum trial, PLA placebo trial, ES effect size, *↑ Significantly greater than PLA, *↓ Significantly less than PLA, ↔ No different between trials.

## Data Availability

All relevant materials are presented in the present manuscript.

## Supplementary Materials

The following supporting information can be downloaded at: https://www.mdpi.com/article/10.3390/nu16213611/s1, Table S1. Quality Assessment of Controlled Intervention Studies (QACIS) of the included articles. Table S2. Quality Assessment of Controlled Intervention Studies (QACIS) Criteria. Table S3. Description of the characteristics of the studies.
