# Supplementary material for: Effects of Caffeinated Chewing Gum on Exercise Performance and Physiological Responses: A Systematic Review
Source: Nutrients. 2024 Oct 24;16(21):3611. doi: 10.3390/nu16213611 (PMC11547772; doi:10.3390/nu16213611)
Supplement: Supplementary file 1 [file nutrients-16-03611-s001.zip › Table S3. Description of the characteristics of the studies.pdf]

Table S3. Description of the characteristics of the studies

Author (Year)	Study Design	Participant Information N (M/F)	Caffeine dose	Chewing Duration	Timing	Measurements	Main Outcome
Paton et al. (2010)	DB<br>RA<br>CR<br>PL<br>CB	n=9 (9/0)<br>Well-trained competitive cyclist<br>Age: 24.1 ± 7.3 years<br>Height: 182 ± 6 cm<br>Weight: 79.3 ± 10.6 kg<br>VO2max: 62.5 ± 5.4 mL/kg/min<br>PA: 351 ± 32 W<br>Caffeine intake: < 300 mg/day	240 mg	5 min	during a 10-min recovery period after completion of the second exercise set.	4 sets*5 *30-s maximal effort sprints<br>Testosterone concentration (Salivary)<br>Cortisol concentrations (Salivary)	Mean Power output: declined by 5.8 ± 4.0% in the placebo and 0.4 ± 7.7% in the caffeine<br>*↓ fatigue (5.4%, ES: 0.25 ± 0.16)<br>*↑ testosterone concentration (12%, ES: 0.50)<br>*↓ cortisol concentration (21%, ES: -0.30)
Bellar et al. (2011)	DB<br>RA<br>PL<br>RM	n=10 (5/5)<br>Healthy college Age<br>Age: 23.0 ± 3.9 / 24.2 ± 1.5 years<br>Height: 172.3 ± 9.9 / 166.2 ± 9.8 cm<br>Weight: 86.2 ± 12.9 / 70.5 ± 9.4 kg<br>Body fat: 14.5 ± 5.2 / 25.2 ± 3.7 %<br>Caffeine intake: None report	100 mg	5 min	immediately	Grip to Exhaustion Task;<br>Forearm muscle pain;<br>RPE	⇔ Grip to exhaustion (CAF: 104.98 ± 57.95, PLA: 99.85 ± 78.39, p=0.786, ES: 0.009)<br>*↓ forearm muscle pain (CAF: 3.45 ± 2.95, PLA: 4.84 ± 2.92, p<0.001, phi: 0.377)<br>⇔ RPE (CAF: 13.45 ± 3.23, PLA: 13.32 ± 4.15, p=0.411, phi: 0.275)
Bellar et al. (2012)	DB<br>RA<br>CR	n=9 (4/5)<br>college shot putter<br>Age: 20.5 ± 2.1 / 19.6 ± 1.9 years<br>Height: 1.82 ± 0.07 / 1.73 ± 0.07 m<br>Weight: 115 ± 13 / 98 ± 28<br>Caffeine intake: 73±97 mg	100 mg	5 min	immediately	simple reaction time task;<br>shot put performance	*↓ reaction time (pre-CAF: 0.306 ± 0.05 s, PLA: 0.317 ± 0.06 s, post-CAF: 0.316 ± 0.08 s, PLA: 0.323 ± 0.06 s, p<0.05, ES>0.73)<br>*↑ first throw test (CAF: 9.62 ± 1.71m, PLA: 9.05 ± 1.69m, p=0.050, ES: 0.996)<br>*↑ first group of three throws (p=0.067, ES:0.359)
Ryan et al. (2012)	DB<br>RA<br>PL<br>RM<br>CR	n=8 (8/0)<br>College-aged physical active<br>Age: 26 ± 4 years<br>Weight: 85 ± 15 kg<br>VO2max: 45.5 ± 5.7 mL/kg/min<br>Caffeine intake: < 300 mg/day	200 mg	5 min	one of three time points: 35 min before test; 5 min before test; 15 min after test;	TTE at 85% VO2max;<br>Blood sample<br>Oxygen consumption<br>RER<br>HR<br>glucose<br>lactate<br>RPE<br>perceived leg pain	⇔ TTE at 85% VO2max (p=0.980)<br>⇔ serum-free fatty acid (time: p>0.005)<br>⇔ plasma epinephrine concentration (time: p<0.005)<br>⇔ plasma norepinephrine concentration (time: p<0.005)<br>⇔ Oxygen consumption (time: p<0.001)<br>⇔ RER (time: p<0.005)<br>⇔ HR (time: p<0.005)<br>⇔ glucose (time: p>0.005)<br>⇔ lactate (time: p<0.005)<br>⇔ RPE (time: p<0.005)<br>⇔ perceived leg pain (time: p<0.005)
Bashafaat et al. (2013)	DB<br>QE	n=15 (15/0)<br>time trial cyclist<br>Age: 25.2 ± 2.7 years<br>Height: 170.5 ± 5.9 cm<br>Weight: 68.3 ± 4.5 kg<br>Body fat: 13 ± 2.7 %<br>Caffeine intake: None report	180 mg<br>300 mg	5 min	three time points<br>180 mg at 30 minutes before;<br>300 mg at 5 minutes before;<br>immediately after the 1 and 4-km cycling	1 km or 4 km time trial performance<br>Blood glucose<br>Blood lactate	⇔ time trial performance for 1 km or 4 km (p>0.05)<br>⇔ blood glucose (p>0.05)<br>⇔ blood lactate (time: p<0.05)

Table S3. Description of the characteristics of the studies

Author (Year)	Study Design	Participant Information N (M/F)	Caffeine dose	Chewing Duration	Timing	Measurements	Main Outcome
Ryan et al. (2013)	PL<br>RM<br>CR	n=8 (8/0)<br>College-Aged trained cyclist<br>Age: 25 ± 5 years<br>VO2max: 50 ± 5 mL/kg/min<br>Caffeine intake: < 300 mg/day	300 mg	5 min	one of three time points:<br>120 min before test;<br>60 min before test;<br>5 min before test	Cycling time trial<br>RER<br>RPE<br>HR<br>Perceived leg pain (LP)<br>blood sample	*↓ finish time at trial ingested 5 min before test (CAF: 38.7 ± 1.2, PLA: 40.7 ± 1.2, p=0.023)<br>⇔ RER (time: p<0.035)<br>⇔ RPE (time: p<0.035)<br>⇔ HR (time: p<0.035)<br>⇔ LP (time: p<0.035)<br>⇔ β-endorphin (time: p<0.035)<br>*↑ Plasma caffeine concentration during the cycling time trial at trial ingested 5 min before test (p=0.046) not in -120 or -60 trial
Lane et al. (2014)	DB<br>LS	n=24 (12/12)<br>competitive cyclists or triathletes<br>Age: 31 ± 7/28 ± 6 years<br>Height: 180.8 ± 6.1/169.1 ± 8.0 cm<br>Weight: 73.4 ± 6.8/62.1 ± 8.9 kg<br>Maximal aerobic power (MAP): 459.4 ± 31.1/327.1 ± 32.3 W<br>VO2peak: 71.6 ± 4.6/59.9 ± 5.1 mL/kg/min<br>Caffeine intake: None report	3 mg/kg	10 min	2 mg/kg BM:<br>40 min before the time trial<br>1 mg/kg BM:<br>10 min before the time trial	London Olympic Games cycling time trial<br>HR<br>RPE<br>Blood sample	*↑ Blood caffeine concentration within 30 min of ingestion (CAF: 10.0 ± 3.80 μmol/L)<br>*↑ Peak caffeine concentration (CAF: 17.2 ± 5.5 μmol/L)<br>*↑ Mean power output (CAF: 260 ± 58 W, PLA: 250 ± 57 W, p<0.001)(improvement 4.0 ± 1.7 %MAP, p<0.001)<br>*↓ Time trial finish time (male: 1.3%, p<0.001, female: 1.6%, p<0.001)<br>⇔ RPE (CAF: 17 ± 0.8/17 ± 1.0, PLA: 17 ± 0.8/17 ± 1.2)<br>⇔ HR (CAF: 172 ± 10/174 ± 9, PLA: 167 ± 11/171 ± 8)
Paton et al. (2015)	DB<br>RA<br>CR<br>PL<br>CB	n=20 (10/10)<br>trained cyclist<br>Age: 30 ± 10 years<br>Height: 172 ± 7 cm<br>Weight: 69 ± 10 kg<br>VO2max: 4.2 ± 0.7 L/min<br>Caffeine intake: < 300 mg/day	male:<br>300 mg<br>female:<br>200 mg	5 min	immediately following the first maximal sprint at 10 km	3 lap*10-km<br>sprint at last 0.2 km in each lap<br>HR<br>RPE<br>VO2<br>blood lactate	⇔ first 20-km performance or physiological<br>*↑ mean and sprint power output during the third lap<br>*↑ HR during the third lap<br>*↑ Blood lactate during the third lap (CAF: 5.2 ± 2.3, PLA: 4.7 ± 2.2 mmol/L, ES: ~0.2)
Oberlin-Brown et al. (2016)	DB<br>CR<br>RM	n=11 (11/0)<br>endurance-trained cyclists or triathletes<br>Age: 32.2 ± 7.5 years<br>Weight: 74.3 ± 6.8 kg<br>Sum of 8 skinfolds: 67.8 ± 13.3 mm<br>VO2peak: 60.2 ± 4.0 mL/kg/min<br>Maximum aerobic power: 331 ± 34 W<br>Caffeine intake: None report	4*50 mg	5 min	every 25% time trial	20 km time trial (time/power output/cadence)<br>blood glucose<br>blood lactate<br>HR	⇔ Time trial performance (CAF: 32:20 ± 1:57, PLA: 32:27 ± 1:57 m:s, 0.4 ± 2.2% difference between CAF and PLA)<br>⇔ Mean power output (CAF: 273 ± 40, PLA: 270 ± 37 W, ES: 0.03)<br>*↑ Mean power output for 10-15 km (CAF: 263 ± 39, PLA: 259 ± 35 W, ES: 0.09)<br>*↑ Mean power output for 15-20 km (CAF: 284 ± 42, PLA: 273 ± 41 W, ES: 0.24)<br>⇔ Average HR (CAF: 176 ± 11, PLA: 163 ± 13)<br>⇔ Maximal HR (CAF: 176 ± 10, PLA: 164 ± 11)<br>*↑ Blood glucose change (ES: -0.44 ± 0.31)<br>*↑ Blood lactate during post-20-km time trial (ES: 0.90 ± 1.09)

Table S3. Description of the characteristics of the studies

Author (Year)	Study Design	Participant Information N (M/F)	Caffeine dose	Chewing Duration	Timing	Measurements	Main Outcome
Siahpoosh et al. (2016)	DB	n=10 (10/0)<br>middle-distance runners<br>Age: 24.8 ± 1.6 years<br>Height: 174.5 ± 3.9 cm<br>Weight: 65.5±4.7 kg<br>body fat percentage: 13 ± 2.7 %<br>Caffeine intake: None report	180 mg<br>300 mg	5 min	30 min before test<br>5 min before<br>immediately after test	Glucose<br>Blood lactate	⇔ blood lactate<br>⇔ blood glucose<br>⇔ 800 m run performance (CAF180: 2.03 ± 0.41, CAF300: 2.02 ± 0.29, PLA: 2.04 ± 0.43 min; F=11.32, p=0.095)<br>⇔ 1500 m run performance (CAF180: 4.17 ± 0.18, CAF300: 4.18 ± 0.36, PLA: 4.22 ± 0.23 min; F=6.098, p=0.108)
Thomas et al. (2017)	DB<br>RM<br>PL<br>RA	n=20 (13/7)<br>untrained healthy adults<br>A/A homozygotes n=11 (7/4)<br>C allele carrier n=9 (6/3)<br>Age: 25.5 ± 3.5 years<br>BMI: 26.2 ± 4.6 kg/m2<br>VO2peak: 32.2 ± 6.4 ml/kg/ min<br>Caffeine intake: < 300 mg/day	300mg	5 min	5 min before test	HRV at baseline, 0-5 min post exercise, 5-10 min post exercise (cycle d for 15 min at 75% VO2 peak)<br>respiratory rate (RRate)	*↓ ApEn for the A/A group during 5-10 min post exercise (CAF: 0.8 ± 0.1 ,PLA: 0.9 ± 0.1, p=0.013, d=0.96)<br>*↓ The rate of ApEn recovery for A/A group (CAF: 0.3 ± 0.2,PLA: 0.4 ± 0.2 ms, p=0.04; d=0.48)<br>CAF did not impaired HRV indexes recovery after exercise.
Evans et al. (2018)	DB<br>RA<br>CR	n=18 (18/0)<br>team sport athletes<br>Age: 21.2 ± 1.1 years<br>Height: 1.78 ± 0.06 m<br>Weight: 80.4 ± 6.6 kg<br>body fat: 14.7 ± 3.9%<br>Fat-free mass: 68.7 ± 6.0 kg<br>Caffeine intake: 22±12 to 231±88 mg/day	200 mg	10 min	5 min before test	40 m maximal shuttle run test (MST)	⇔ Sprint total time (CAF: 87.5 ± 3.1, PLA: 87.8 ± 3.0 s, p=0.214, ES: 0.10)<br>⇔ Fastest sprint time (CAF: 8.33 ± 0.23, PLA: 8.33 ± 0.20 s, p=0.879, ES: 0.02)<br>⇔ slowest sprint time (CAF: 9.06 ± 0.39, PLA: 9.10 ± 0.43 s, p=0.452, ES: 0.10)<br>*↑ blood lactate after MST (CAF: 11.2 ± 2.3, PLA: 10.3 ± 2.6 mM, p=0.035, ES: 0.36)<br>⇔ Overall sprint performance decrement (CAF: 5.00 ± 2.84, PLA: 5.43 ± 2.68 %, p=0.209, ES: 0.16)<br>*↓ Sprint performance decrement for low habitual participants (CAF: 5.33 ± 3.12, PLA: 6.53 ± 2.91 %, p=0.049, ES: 0.33)<br>⇔ Sprint performance decrement for moderate-to-high habitual participants (CAF: 3.98 ± 2.57, PLA: 3.80 ± 1.79 %, p=0.684, ES: 0.08)<br>⇔ HR (time, p<0.001; trial, p=0.366; interaction, p=0.882)
Ranchordas et al. (2018)	DB<br>RA<br>CR<br>CB	n=10 (10/0)<br>university-standard soccer players<br>Age: 19 ± 1 years<br>Height: 1.80 ± 0.10 m<br>Weight: 75.5 ± 4.8 kg<br>Soccer experience: 4 ± 0.9 years<br>Caffeine intake: None report	200 mg	5 min	5 min before test	CMJ<br>20 m sprint test<br>YO-YO IR1 test	*↑ CMJ (CAF: 47.1 ± 3.4, PLA: 46.1 ±3.2 cm, p=0.008, ES: 0.30; enhancee by 2.2%)<br>⇔ 20 m sprint test (CAF: 3.2 ± 0.3, PLA: 3.1 ±0.3 s, p=0.567, ES: 0.33)<br>*↑ Covered distance in YO-YO IR1 test (CAF: 1754 ± 156, PLA: 1719 ± 139 m, p=0.016, ES: 0.24; enhancee by 2 %)
Ranchordas et al. (2019)	DB<br>RA<br>CR<br>PL	n=17 (17/0)<br>competitive university-standard rugby players<br>Age: 20.4 ± 1.2 years<br>Height: 179.4 ± 6.2 cm<br>Weight: 85.6 ± 6.3 kg<br>Caffeine intake: None report	200 mg	5 min	immediately	CMJ<br>Illinois agility test<br>6*30 m repeat sprint test<br>YO-YO IR2 test<br>RPE<br>Blood lactate	*↑ CMJ (CAF: 43.7 ± 7.6; PLA: 42.2 ± 6.2 cm, p=0.044, ES: 0.22; enhanced by 3.6 %)<br>⇔ Illinois agility test (CAF: 16.22 ± 1.08; PLA: 15.88 ± 1.09 s, p=0.271, ES: -0.31)<br>⇔ 6*30 m repeated sprints performance (trial: p=0.341; time: p<0.001; trial*time: p=0.256)<br>*↓ Fatigue index (CAF: 102.2 ± 0.9; PLA: 103.3 ± 1.2 %, p=0.001, ES: 1.03)<br>*↑ Covered distance in YO-YO IR2 test (CAF: 426 ± 105; PLA: 372 ± 91 m, p=0.010, ES: 0.55; enhanced by 14.5 %)<br>⇔ Blood lactate (CAF: 14.4 ± 3.0; PLA: 13.2 ± 2.5 mmol/L, p=0.075, ES: 0.43)<br>⇔ HR (CAF: 173 ± 7; PLA: 169 ± 14 beat/min, p=0.204, ES: 0.264)

Table S3. Description of the characteristics of the studies

Author<br>(Year)	Study<br>Design	Participant Information<br>N (M/F)	Caffeine<br>dose	Chewing<br>Duration	Timing	Measurements	Main Outcome
Venier et al.<br>(2019)	DB<br>RA<br>CR	n=19 (19/0)<br>healthy adult<br>Age: 24 ± 5 years<br>Height: 183 ± 5 cm<br>Weight: 83 ± 10 kg<br>Caffeine intake: 67±85 mg/day	300 mg	10 min	immediately	CMJ<br>SJ<br>Isokinetic strength and power<br>Bench press<br>Rowing ergometer test	*↑ CMJ (CAF: 36.4 ± 6.2; PLA: 34.8 ± 5.8 cm, p<0.001, ES: 0.27)<br>*↑ SJ (CAF: 31.9 ± 6.0; PLA: 30.8 ± 5.3 cm, p=0.023, ES: 0.21)<br>*↑ Peak torque at angular velocity of 60°/s for knee extensor (CAF: 245.0 ± 43.3; PLA: 236.6 ± 36.2 Nm, p=0.048, ES: 0.21)<br>*↑ Average power at angular velocity of 60°/s for knee extensor (CAF: 180.0 ± 34.1; PLA: 172.1 ± 29.1 W, p=0.031, ES: 0.25)<br>*↑ Peak torque at angular velocity of 60°/s for knee flexion (CAF: 142.7 ± 25.5; PLA: 137.1 ± 25.4 Nm, p=0.040, ES: 0.22)<br>⇔ Average power at angular velocity of 60°/s for knee flexion (CAF: 111.3 ± 21.3; PLA: 109.5 ± 21.4 W, p=0.320, ES: 0.09)<br>⇔ Peak torque at angular velocity of 180°/s for knee extensor (CAF: 170.2 ± 28.7; PLA: 164.4 ± 23.8 Nm, p=0.073, ES: 0.22)<br>*↑ Average power at angular velocity of 180°/s for knee extensor (CAF: 322.1 ± 59.1; PLA: 306.2 ± 48.4 W, p=0.035, ES: 0.30)<br>*↑ Peak torque at angular velocity of 180°/s for knee flexion (CAF: 107.6 ± 17.6; PLA: 101.7 ± 19.9 Nm, p=0.021, ES: 0.31)<br>⇔ Average power at angular velocity of 180°/s for knee flexion (CAF: 196.6 ± 41.9; PLA: 188.8 ± 48.9 W, p=0.265, ES: 0.17)<br>*↑ Average power at angular velocity of 180°/s for knee extensor<br>*↑ Bench press velocity at 50% 1 RM (CAF: 0.85 ± 0.08; PLA: 0.82 ± 0.09 m/s, p=0.044, ES: 0.30)<br>*↑ Bench press velocity at 75% 1 RM (CAF: 0.57 ± 0.07; PLA: 0.54 ± 0.06 m/s, p=0.005, ES: 0.44)<br>*↑ Bench press velocity at 90% 1 RM (CAF: 0.38 ± 0.07; PLA: 0.35 ± 0.07 m/s, p=0.002, ES: 0.43)<br>*↑ Peak power output on the rowing ergometer test (CAF: 667.5 ± 78.5; PLA: 635.9 ± 68.7 W, p=0.006, ES: 0.41)
Daneshfar et al.<br>(2020)	DB<br>RA<br>CR<br>PL	n=14 (14/0)<br>male motocross riders<br>Age: 20.0 ± 3.3 years<br>Height: 1.78 ± 0.04 m<br>Weight: 72 ± 4 kg<br>Caffeine intake: None report	300 mg	NA	NA	bicycle motocross time trial	*↑ Time trial performance (p=0.001, ES: 0.71)<br>*↑ Peak power to weight ratio (p=0.001, ES: 0.79)<br>*↑ Maximal power to weight ratio (p-0.001, ES: 0.80)<br>*↓ RPE (CAF: 6.6 ± 1.3, PLA: 7.2 ± 1.7, p=0.001, ES: 0.64)
Russell et al.<br>(2020)	DB<br>RA<br>CR<br>PL<br>CB	n=14 (14/0)<br>professional academy rugby player<br>Age: 18 ± 1 years<br>Height: 1.83 ± 0.07 m<br>Weight: 98.6 ± 10.9 kg<br>Caffeine intake: 191 ± 138 mg/day	400 mg	5 min	10 min before test	Repeated sprint testing<br>Blood lactate<br>Salivary testosterone<br>Salivary cortisol<br>Simple reaction time test<br>Stroop test	⇔ Sprint performance (trial*time: p=0.995; time: p<0.001)<br>⇔ Blood lactate (trial*time: p=0.778; time: p<0.001)<br>*↑ Salivary testosterone before RSSA2(第二次反覆衝刺前)(trial*time, p<0.001; CAF>PLA, p<0.001 increase 70%; time: p<0.001)<br>⇔ Salivary cortisol concentration (time: p<0.001; trial: p=0.307)<br>⇔ Simple reaction time test (trial*time: p=0.510; time: p=0.058)<br>⇔ Stroop test reaction time on incongruent (time*trial: p=0.299; time: p=0.845)<br>⇔ Stroop test reaction time on congruent (time*trial: p=0.495; time: p=0.362)
Whalley et al.<br>(2020)	DB<br>RA<br>CR	n=14<br>amateur runner<br>Caffeine intake: None report	3-4.5<br>mg/kg	NA	15 min before test	5 km running time trial<br>HR<br>RPE	*↑ 5 km running time trial performance (Gum: 0.9% ± 1.4%, p>0.005)<br>⇔ HR<br>⇔ RPE

Table S3. Description of the characteristics of the studies

Author (Year)	Study Design	Participant Information N (M/F)	Caffeine dose	Chewing Duration	Timing	Measurements	Main Outcome
Dittrich et al. (2021)	DB<br>RA<br>PL<br>RM	n=12 (12/0)<br>trained endurance runners<br>Age: 31.3 ± 6.4 years<br>Height: 175.2 ± 6.2 cm<br>Weight: 70.5 ± 6.6 kg<br>Bodt fat: 9.4 ± 2.7%<br>VO2max: 62.0 ± 4.2 ml/kg/min<br>Caffeine intake: None report	300 mg	5 min	NA	TTE at 50% maximal aerobic speed<br>Voluntary activation level (VA%) during MVCs<br>Neuromuscular function test<br>Muscle activity during MVC<br>HR<br>RPE	*↑ TTE at 50% maximal aerobic speed (CAF: 40.60 ± 8.53; PLA: 33.23 ± 7.41 min, p<0.001)<br>*↑ Total distance covered (CAF: 10.36 ± 2.19; PLA: 8.45 ± 1.73 km, p<0.001)<br>⇔ delta blood lactate (comparison before and after)(CAF: 1.99 ± 0.61; PLA: 1.55 ± 0.88 mmol/L, p=0.08)<br>⇔ Mean RPE (CAF: 6.2 ± 1.2; PLA: 6.2 ± 1.0, p=0.69)<br>*↓ RPE at exhaustion time (CAF: 8.8 ± 1.2; PLA: 10.4 ± 0.5, p<0.01)<br>⇔ Neuromuscular function (time*trial: p>0.05)<br>*↓ Reduction of MVC after exercise (CAF: pre 279.9 ± 56.5; post 244.0 ± 46.3; PLA: pre 280.4 ±52.8; post 237.7 ± 50.5 N/m)<br>⇔ Muscle activity<br>⇔ HR (time: p<0.01)<br>⇔ VE (time: p<0.01)<br>⇔ RER (time: p<0.01)<br>⇔ Electrical response peak twitch (time: p<0.01)<br>⇔ Maximal voluntary activation (time: p<0.01)<br>⇔ M-wave duration (CAF: pre 9.3 ± 1.6; post 8.8 ± 1.3; PLA: pre 9.7 ±1.3 ; post 8.8 ± 1.0 m; time: p<0.05)<br>⇔ M-wave amplitude (CAF: pre 15.9 ± 5.6; post 15.4 ± 6.3; PLA: pre 16.5 ± 3.4; post 16.5 ± 4.2 mV)<br>⇔ VL muscle RMS/M ratio (time: p<0.05)<br>⇔ Torque (time: p<0.01)<br>⇔ Twitch potentiated (time: p<0.01)
Filip-Stachnik et al. (2021)	DB<br>RA<br>CR<br>PL	n=9 (9/0)<br>healthy experienced judoists<br>Age: 23.7 ± 4.4 years<br>Height: 174.3 ± 7.4 cm<br>Weight: 73.5 ± 7.4 kg<br>Body fat: 11.1 ± 4.0 %<br>Training experience: 3.1 ± 1.3 years<br>Caffeine intake: 3.1 ± 1.3 mg/kg BM	200 mg<br>400 mg	5 min	15 min before test	Special Judo Fitness Test<br>SJFT Index<br>Blood lactate<br>RPE<br>HR	⇔ Total number of SJFT throws (CAF200: 62.22 ± 4.32; CAF400: 60.22 ±4.08; PLA: 59.66 ±4.15; (trial*time: p=0.0.447; trial, p=0.063; time, p=0.111)<br>⇔ SJFT Index (trial*time: p=0.961; trial, p=0.099; time, p=0.193)<br>⇔ RPE (trial*time: p=0.896; trial, p=0.538; time, p=0.153)<br>⇔ Blood lactate (trial*time: p=0.223; trial, p=0.098; time, p=0.869)<br>⇔ HR (trial*time: p=0.782; trial, p=0.525; time, p=0.971)
Mask et al. (2021)	DB<br>RA<br>CR<br>CB	n=16 (0/16)<br>recreationally active college females<br>Age: 21 ± 2.8 years<br>Height: 1.66 ± 0.06 m<br>Weight: 63.9 ± 11.2 kg<br>Body fat: 25.5 ± 6.0 %<br>VO2peak: 21.8 ± 2.9 ml/kg/min<br>Caffeine intake: 23 ± 20 to 195 ± 93 mg/day	300 mg	NA	15 min before test	Arm ergometer cadence for RPE production trial (4 or 7)<br>Likert scale	⇔ CAD at RPE 4 (CAF: 37.7 ± 1.6; PLA: 37.6 ± 1.6 rev/min, p>0.05)<br>⇔ CAD at RPE 7 (CAF: 42.9 ± 1.6; PLA: 41.2 ± 1.7 rev/min, p>0.05)<br>⇔ HR (trial, p>0.05)<br>⇔ RER (trial, p>0.05)<br>⇔ VO2 at RPE 7 (trial, p>0.05)<br>*↑ restlessness (CAF: 3.5 ± 1.9; PLA: 2.2 ± 2.3, p=0.03; ES=0.64)<br>⇔ fatigue, mood, nervousness, tremors, distress (p>0.05)

Table S3. Description of the characteristics of the studies

Author (Year)	Study Design	Participant Information N (M/F)	Caffeine dose	Chewing Duration	Timing	Measurements	Main Outcome
Whalley et al. (2021)	RA<br>CR	n=14 (10/4)<br>experienced runners<br>Age: 40 ± 8 years<br>Height: 177 ± 11 cm<br>Weight: 69 ± 11 kg<br>Caffeine intake: ten habitual caffeine users ; four non-habitual caffeine users	65 kg ↓ :<br>200 mg<br>65 kg ↑ :<br>300 mg	NA	15 min before test	5 km running time trial<br>HR<br>RPE	⇔ post-run urinary caffeine or paraxanthine concentration
Filip-Stachnik et al. (2022)	DB<br>RA<br>CR<br>PL<br>CB	n=12 (0/12)<br>volleyball players<br>Height: 177 ± 11 cm<br>Weight: 69 ± 11 kg<br>Caffeine intake: 2.7 ± 2.1 mg/kg/day	400 mg	5 min	15 min before test	Attack jump<br>Block jump<br>Game assessment<br>HR	*↑ Attack jump height (p=0.024, pre-game CAF: 47.2 ± 7.3, PLA: 46.0 ± 7.9 cm, p=0.032, post-game: CAF: 47.5 ± 7.5, PLA: 46.3 ± 8.3 cm, p=0.022, ES: 0.15)<br>⇔ Block jump height (pre-game CAF: 33.0 ± 4.5, PLA: 32.6 ± 5.7 cm, post-game: CAF: 34.7 ± 6.2, PLA: 34.8 ± 6.4 cm, p=0.724)<br>⇔ Mean HR (CAF:136 ± 10; PLA: 134 ± 12 bpm, p=0.724)<br>⇔ Peak HR (CAF:176 ± 10; PLA: 178 ± 11 bpm, p=0.794)<br>⇔ Number of jumps during the game (CAF:52 ± 13; PLA: 47 ± 15 jumps, p=0.273)<br>⇔ Volleyball specific skill during the game (total points, total errors, service points, service errors, reception errors, negative reception, positive reception, perfect reception, and blocking points, p>0.05)
Kaszuba et al. (2022)	DB<br>RA<br>CR	n=12 (9/3)<br>volleyball players<br>Age: 23 ± 3 years<br>Height: 188 ± 8 cm<br>Weight: 85.9 ± 11.2 kg<br>BMI: 24.4 ± 1.7 kg/m2<br>Training experience: 9 ± 4 years<br>Caffeine intake: 2.7±2.2 mg/kg/day	male:<br>300 mg<br>female:<br>200 mg	5 min	15 min before test	CMJ<br>SJ<br>Attack jump<br>Block jump<br>5 and 10 m sprint<br>modified agility t-test<br>attack and service speed test<br>spike and serve accuracy test	⇔ CMJ (CAF: 51.2 ± 11.2, PLA: 51.0 ± 11.4 cm, p=0.820)<br>⇔ SJ (CAF: 39.1 ± 7.8, PLA: 40.9 ± 9.6 cm, p=0.230)<br>⇔ Attack jump (CAF: 61.4 ± 14.9, PLA: 62.4 ± 13.9 cm, p=0.342)<br>⇔ Block jump (CAF: 48.4 ± 10.6 , PLA: 48.4 ± 11.6 cm, p=0.995)<br>⇔ 5 m sprint (CAF: 0.95± 0.11, PLA: 0.95 ± 0.11 s, p=1.000)<br>⇔ 10 m sprint (CAF: 1.69 ± 0.12, PLA: 1.68 ± 0.13 s, p=0.619)<br>⇔ Agility t-test (CAF: 9.44 ± 0.69, PLA: 9.45 ± 0.77 s, p=0.952)<br>⇔ Standing attack speed (CAF: 82 ± 11, PLA: 79 ± 12 km/h, p=0.274)<br>⇔ Attack speed (CAF: 85 ± 14, PLA: 81 ± 13 km/h, p=0.119)<br>⇔ Service speed (CAF: 88 ± 14, PLA: 86 ± 13 km/h, p=0.254)<br>*↑ Attack accuracy (CAF: 18 ± 3, PLA: 15 ± 4 points, p=0.023, ES: 0.85)<br>⇔ Serve accuracy (CAF: 12 ± 4, PLA: 10 ± 3 points, p=0.140)
Sargent et al. (2022)	DB<br>RM<br>PL	n=18(9/9)<br>healthy, college-Aged, physically active adults<br>Age: 22.1 ± 2.6<br>Weight: 80.0 ± 10.0<br>BMI: 26.9 ± 4.3<br>VO2max: 45.1 ± 8.3<br>Caffeine intake: < 300 mg/day	200 mg	5 min	5 min before test	HRV	*↑ SDNN<br>*↑ LF<br>*↑ HF<br>*↑ SD1<br>*↑ RMSSD

Table S3. Description of the characteristics of the studies.

Author (Year)	Study Design	Participant Information N (M/F)	Caffeine dose	Chewing Duration	Timing	Measurements	Main Outcome
Chen et al. (2023)	SB<br>RA<br>CR	n=19(19/0)<br>healthy adult<br>Age: 22.5 ± 3.5 years<br>Height: 176.2 ± 6.5 cm<br>Weight: 78.8 ± 13.2 kg<br>Caffeine intake: 62.55 ± 94.01 mg	200mg	5 min	10 min before test	Romanian deadlift on flywheel inertial device<br>HR<br>RPE	*↑ Peak concentric power (p=0.016, ES: 0.44)<br>*↑ Peak eccentric power (p=0.005, ES: 0.55)<br>*↑ Average power (p=0.013, ES: 0.43)<br>⇔ Average force (p=0.063, ES: 0.50)<br>*↑ Total work (p=0.026, ES: 0.28)<br>⇔ HR (CAF: 132.9 ± 18.7, PLA: 127.9 ± 12.7 bpm, p=0.143)<br>⇔ RPE (CAF: 11.7 ± 2.4, PLA: 12.0 ± 2.5, p=0.266)
Pirmohammadi et al. (2023)	DB<br>RA<br>CR<br>PL	n=18 (0/18)<br>table tennis player<br>Age: 20.50 ± 3.05 years<br>Height: 165.16 ± 5.00 cm<br>Weight: 56.83 ± 6.50 kg<br>BMI: 20.83 ± 2.44 kg/m2<br>Caffeine intake: None report	65 kg ↓ : 200 mg<br>65 kg ↑ : 300 mg	10 min	immediately	Edgren's agility test<br>Hand movement speed test<br>Alternate Hand Wall Toss Test (HWTT)<br>Movement speed test<br>Sargent's jump test (Lower body explosive power)<br>medicine ball throwing (upper body explosive power)<br>handgrip strength<br>Service Test<br>Backhand-push Test<br>Counter Test<br>Forehand Drive<br>Cognitive test<br>(Functional tests, Skill tests, Cognitive tests)	*↑ Edgren's agility test (CAF: 24.38 ± 2.19, PLA: 23.22 ± 2.41 score, p=0.002)<br>*↑ hand movement speed (CAF: 11.08 ± 1.27, PLA: 12.19 ± 1.43 n, p<0.001)<br>*↑ movement speed (CAF: 3.74 ± 0.22, PLA: 4.16 ± 4.0 s, p=0.001)<br>⇔ Accuracy in eye-hand coordination test (CAF: 28 ± 1.45, PLA: 26.88 ± 2.21 n, p=0.091)<br>*↑ Sargent's jump test (CAF: 1865.11 ± 452.00, PLA: 1689.55 ± 49.68 Watt, p=0.001)<br>⇔ Handgrip strength (CAF: 55.27 ± 8.30, PLA: 53.05 ± 7.5 kg, p=0.311)<br>*↑ Cognitive test (CAF: 22.44 ± 1.5, PLA: 2.11 ± 1.84 n, p<0.001)<br>⇔ Accuracy of service (CAF: 38.11 ± 4.82, PLA: 36.33 ± 4.02 score, p=0.211)<br>⇔ Accuracy of forehand drive (CAF: 16.11 ± 2.58, PLA: 15.72 ± 1.99 score, p=1.000)<br>⇔ Backhand-push performance (CAF: 28.05 ± 1.25, PLA: 28.30 ± 0.94 score, p=0.318)<br>⇔ Counter performance (CAF: 26.66 ± 1.87, PLA: 26.83 ± 1.33 n, p=0.710)<br>⇔ Explosive power of upper body muscle (CAF: 448.33 ± 61.59, PLA: 438.66 ± 44.76 cm, p=0.192)
Yildirim et al. (2023)	DB<br>RA<br>CR<br>CB	n=14 (14/0)<br>highly trained soccer player<br>Age: 22 ± 2 years<br>Height: 180.0 ± 6.8 cm<br>Weight: 74.2 ± 7.1 kg<br>Body fat percent: 11.4 ± 3.9%<br>Training experience: 11 ± 1 years<br>Caffeine intake: 358.9 ± 292.4 mg/day	100mg<br>200mg	5 min	15 min before test	handgrip strength<br>quadriceps strength<br>hamstring strength<br>ball-kicking speed<br>15-s CMJ tests	*↑ quadriceps strength (MCAF: 53.77 ± 5.77, LCAF: 49.62 ± 8.81, PLA: 49.20 ± 7.20 kg, MCAF>LCAF, p=0.048, ES: 0.55; MCAF>PLA, p=0.032, ES: 0.70)<br>⇔ Hamstring strength (MCAF: 26.81 ± 5.83, LCAF: 28.11 ± 6.12, PLA: 25.66 ± 3.49 kg, p=0.251)<br>⇔ Handgrip strength (MCAF: 48.76 ± 6.53, LCAF: 48.16 ± 6.22, PLA: 47.07 ± 6.82 kg, p=0.145)<br>⇔ Ball-kicking speed (MCAF: 106.36 ± 5.79, LCAF: 107.80 ± 4.34, PLA: 106.74 ± 7.69 km/hr, p=0.658)<br>⇔ Peak jump heights (MCAF: 34.62 ± 5.42, LCAF: 37.48 ± 7.39, PLA: 37.27 ± 4.57 cm, p=0.678)<br>⇔ Mean jump heights (MCAF: 30.20 ± 6.31, LCAF: 32.23 ± 5.55, PLA: 32.43 ± 3.95 cm, p=0.833)<br>⇔ Peak jump power (MCAF: 43.70 ± 6.00, LCAF: 46.12 ± 7.85, PLA: 45.91 ± 6.26 Watt/kg, p=0.377)<br>⇔ Mean jump power (MCAF: 40.04 ± 6.05, LCAF: 41.69 ± 5.74, PLA: 41.86 ± 5.01 Watt/kg, p=0.439)
Cagin et al. (2024)	RA	CAF/PLA<br>Age: 20.5 ± 0.18/21.9 ± 0.55 years<br>Height: 167.6 ± 1.66/182.3 ± 2.17 cm<br>Weight: 58.2 ± 1.8/80.3 ± 4.58 kg<br>Training experience: 9.69 ± 0.79/9.33 ± 0.94 years Caffeine intake: None report	5 mg/kg	NA	15 min before test	Flamingo balance test<br>30-meter sprint tests	⇔ Number of balance error (CAF: 3.38 ± 0.76, PLA: 2.87 ± 0.66, p=0.62)<br>*↓ Reaction time (CAF: 0.17 ± 0.22, PLA: 0.19 ± 0.02 s, p=0.02)<br>⇔ Acceleration (CAF: 3.36 ± 0.06, PLA: 2.89 ± 0.05 s, p=0.69)<br>⇔ Total time (CAF: 5.83 ± 0.09, PLA: 5.06 ± 0.09 s, p=0.43)

Table S3. Description of the characteristics of the studies.

Author (Year)	Study Design	Participant Information N (M/F)	Caffeine dose	Chewing Duration	Timing	Measurements	Main Outcome
Farmani et al. (2024)	DB<br>RA<br>CR<br>PL	n=18 (18/0)<br>table tennis player<br>Age: 21.86 ± 2.40 years<br>Height: 173.80 ± 6.88 cm<br>Weight: 61.81 ± 10.32 kg<br>BMI: 20.39 ± 2.63 kg/m2<br>Caffeine intake: 2.1 mg/kg/day	65 kg ↓ : 200 mg<br>65 kg ↑ : 300 mg	10 min	CAF: immediately<br>CMR: 15/10/5 and before test and before Bruce	medicine ball throwing<br>Sargent's jump tests<br>Bruce test<br>VO2max<br>TTE<br>VO2 at VT1<br>VO2 at RCP (respiratory compensation point)	*↑ TTE (CAF: 12.26 ± 1.30, PLA: 11.58 ± 1.22 min, p<0.001)<br>*↑ VO2 at VT1 (CAF: 25.89 ± 2.27, PLA: 24.26 ± 1.57 L/min, p=0.004)<br>*↑ VO2 at RCP (CAF: 31.35 ± 2.92, PLA: 27.93 ± 2.64 L/min, p<0.001)<br>⇔ VO2max (CAF: 50.11 ± 5.14, PLA: 50.50 ± 5.98 ml/min/kg, p=0.877)<br>⇔ throwing medicine ball (CAF: 6.87 ± 0.92, PLA: 6.57 ± 0.97, p=0.928)<br>⇔ Sargent's jump height (CAF: 43.00 ± 5.32, PLA: 42.00 ± 5.79, p=0.596)
Liu et al. (2024)	DB<br>RA<br>CR	n=15 (15/0)<br>basketball player<br>Age: 20.9 ± 1.0 years<br>Height: 180.9 ± 5.4 cm<br>Weight: 77.2 ± 7.5 kg<br>Training experience: 8.2 ± 0.3 years<br>Caffeine intake: None report	3 mg/kg	10 min	15 min before test	stationary free throw shooting test<br>CMJ<br>Agility t-test<br>20 m linear sprint test<br>squat in the flywheel device<br>RAST	*↑ stationary free throw shooting test (CAF: 79.0 ± 14.3%, PLA: 73.0 ± 9.16%, p=0.012, ES:0.94)<br>⇔ CMJ (p=0.147)<br>⇔ Agility T-test (p=0.571)<br>*↑ 20 m sprint split performance (0-10, p=0.045, ES: 0.94/10-20, p=0.019, ES: 0.70)<br>*↑ 20 m sprint performance (CAF: 2.94 ± 1.12s, PLA: 3.13 ± 0.10s, p<0.001, ES: 1.8)<br>*↑ Average power for squat (p=0.012, ES: 0.41)<br>*↑ Peak concentric power for squat (p=0.013, ES: 0.48)<br>*↑ Peak eccentric power for squat (p=0.028, ES: 0.45)<br>⇔ RAST peak power (CAF: 1354.86 ± 44.2, PLA: 1326.70 ± 75.0, p=0.328)<br>*↑ RAST mini power (CAF: 1234.44 ± 75.7, PLA: 1153.90 ± 35.9, p=0.008, ES: 1.35)<br>⇔ RAST peak power per weight (CAF: 17.92 ± 1.9, PLA: 17.53 ± 1.8, p=0.323)<br>*↑ RAST mini power per weight (CAF: 16.30 ± 2.1, PLA: 15.26 ± 1.6, p=0.011, ES: 0.53)<br>*↓ Fatigue index (CAF: 3.60 ± 1.6%, PLA: 5.21 ± 1.6, p=0.009, ES:1.00)
Lynn et al. (2024)	DB<br>RA<br>CR	n=36 (31/5)<br>recreational runner<br>Age: 33.7 ± 10.7 years<br>BMI: 23.1 ± 2.4 kg/m2<br>Caffeine intake: habitual consumers	300 mg	5 min	30 min before test	5 km parkrun finishing time<br>split time for each 1 km<br>HR<br>RPE (6-20)	*↓ 5 km parkrun finishing time (17.28 s, p=0.01)<br>*↓ RPE (CAF: 15.43, PLA: 16.64, p=0.01)<br>⇔ Split time (p=0.06)<br>⇔ Pacing (p=0.21)<br>Treatment allocation: trial 1: 5/14, trial 2: 8/14

Data are reported as mean ± SD. DB double blinded, RA randomized, RM repeated measure, PL placebo-controlled, CR crossover, QE quasi-experimental, LS Latin square, SB Single-blind, CAF caffeinated chewing gum trial, PLA placebo trial, ES effect size, *↑ Significantly greater than PLA, *↓ Significantly less than PLA, ⇔ No different between trials, TTE time to exhaustion, LP perceived of pain, MAP maximum aerobic power, RPE rating of perceived exertion , HR heart rate, HRV heart rate variability, CMJ countermovement jump, SJ squat jump, TT time trial, RAST running-based anaerobic sprint test PO Peak aerobic power output, IR intermittent recovery, VT1 ventilatory threshold, RCP respiratory compensation point, MST maximal shuttle run test , RSP Repeated sprint performance, RAST running-based anaerobic sprint test, W watt, RDL Romanian deadlift,
