# Supplementary material for: Effects of Caffeinated Chewing Gum on Exercise Performance and Physiological Responses: A Systematic Review
Source: Nutrients. 2024 Oct 24;16(21):3611. doi: 10.3390/nu16213611 (PMC11547772; doi:10.3390/nu16213611)
Supplement: Supplementary file 1 [file nutrients-16-03611-s001.zip › Tables S1 and S2.pdf]

Table S1. Quality Assessment of Controlled Intervention Studies (QACIS) of the included articles

Author (Year)	1	2	3	4	5	6	7	8	9	10	11	12	13	14	total	total/14(%)
Paton et al. (2010)	1	1	1	1	1	1	1	1	1	1	1	0	1	1	13	92.86
Bellar et al.(2011)	1	1	1	1	0	1	1	1	1	1	1	1	1	1	13	92.86
Bellar et al. (2012)	1	1	1	1	0	1	1	1	1	1	1	0	1	1	12	85.71
Ryan et al. (2012)	1	1	1	1	0	1	1	1	1	1	1	0	1	1	12	85.71
Bashafaat et al. (2013)	0	0	1	1	0	1	1	1	1	1	1	0	1	1	10	71.43
Ryan et al. (2013)	1	1	1	1	0	1	1	1	1	1	1	0	1	1	12	85.71
Lane et al. (2014)	0	0	1	1	0	1	1	1	1	1	1	0	1	1	10	71.43
Paton et al. (2015)	1	1	1	1	0	1	1	1	1	1	1	0	1	1	12	85.71
Oberlin-Brown et al. (2016)	0	0	1	1	0	1	1	1	1	1	1	0	1	1	10	71.43
Siahpoosh et al. (2016)	0	0	1	1	0	1	0	0	1	1	1	0	1	1	8	57.14
Thomas et al. (2017)	1	1	1	1	0	1	1	1	1	1	1	0	1	1	12	85.71
Evans et al. (2018)	1	1	1	1	0	1	1	1	1	1	1	0	1	1	12	85.71
Ranchordas et al. (2018)	1	1	1	1	0	1	1	1	1	1	1	1	1	1	13	92.86
Ranchordas et al. (2019)	1	1	1	1	0	1	1	1	1	1	1	0	1	1	12	85.71
Venier et al. (2019)	1	1	1	1	0	1	1	1	1	1	1	1	1	1	13	92.86
Daneshfar et al. (2020)	1	1	1	1	0	1	1	1	1	1	1	0	1	1	12	85.71
Russell et al. (2020)	1	1	1	1	0	1	1	1	1	1	1	0	1	1	12	85.71
Whalley et al. (2020)	1	1	0	0	0	1	1	1	1	1	1	0	1	1	10	71.43
Dittrich et al. (2021)	1	1	1	1	0	1	1	1	1	1	1	0	1	1	12	85.71
Filip-Stachnik et al. (2021)	1	1	1	1	0	1	1	1	1	1	1	1	1	1	13	92.86
Mask et al. (2021)	1	1	1	1	1	1	1	1	1	1	1	1	1	1	14	100

1: meets the criteria; 0: does not meet the criteria

Author (Year)	1	2	3	4	5	6	7	8	9	10	11	12	13	14	total	total/14(%)
Whalley et al. (2021)	1	1	0	0	0	1	1	1	1	1	1	0	1	1	10	71.43
Filip-Stachnik et al. (2022)	1	1	1	1	0	1	1	1	1	1	1	1	1	1	13	92.86
Kaszuba et al. (2022)	1	1	1	1	0	1	1	1	1	1	1	1	1	1	13	92.86
Sargent et al. (2022)	0	0	1	1	0	0	1	1	1	1	1	0	1	1	9	64.29
Chen et al. (2023)	1	1	1	0	0	1	1	1	1	1	1	1	1	1	12	85.71
Pirmohammadi et al. (2023)	1	1	1	1	0	1	1	1	1	1	1	0	1	1	12	85.71
Yildirim et al. (2023)	1	1	1	1	0	1	1	1	1	1	1	1	1	1	13	92.86
Cagin et al. (2024)	1	1	0	0	0	1	1	1	1	1	1	0	1	1	10	71.43
Farmani et al. (2024)	1	1	1	1	0	1	1	1	1	1	1	0	1	1	12	85.71
Liu et al. (2024)	1	1	1	0	0	1	1	1	1	1	1	1	1	1	12	85.71
Lynn et al. (2024)	1	1	1	1	1	1	1	1	1	1	1	1	1	1	14	100

1: meets the criteria; 0: does not meet the criteria

Table S2. Quality Assessment of Controlled Intervention Studies (QACIS) Criteria

	Evaluation item
1	Was the study described as randomized, a randomized trial, a randomized clinical trial, or an RCT?
2	Was the method of randomization adequate (i.e., use of randomly generated assignment)?
3	Was the treatment allocation concealed (so that assignments could not be predicted)?
4	Were study participants and providers blinded to treatment group assignment?
5	Were the people assessing the outcomes blinded to the participants' group assignments?
6	Were the groups similar at baseline on important characteristics that could affect outcomes (e.g., demographics, risk factors, co-morbid conditions)?
7	Was the overall drop-out rate from the study at endpoint 20% or lower of the number allocated to treatment?
8	Was the differential drop-out rate (between treatment groups) at endpoint 15 percentage points or lower?
9	Was there high adherence to the intervention protocols for each treatment group?
10	Were other interventions avoided or similar in the groups (e.g., similar background treatments)?
11	Were outcomes assessed using valid and reliable measures, implemented consistently across all study participants?
12	Did the authors report that the sample size was sufficiently large to be able to detect a difference in the main outcome between groups with at least 80% power?
13	Were outcomes reported or subgroups analyzed prespecified (i.e., identified before analyses were conducted)?
14	Were all randomized participants analyzed in the group to which they were originally assigned, i.e., did they use an intention-to-treat analysis?
